# Supplementary material for: Disordered enthalpy–entropy descriptor for high-entropy ceramics discovery
Source: Nature. 2024 Jan 3;625(7993):66–73. doi: 10.1038/s41586-023-06786-y (PMC10764291; doi:10.1038/s41586-023-06786-y)
Supplement: Supplementary file 1 — Supplementary Tables 1–3 and Discussions 1–3. [file 41586_2023_6786_MOESM1_ESM.pdf]

nature portfolio

https://doi.org/10.1038/s41586-023-06786-y

Supplementary information

Disordered enthalpy–entropy descriptor for high-entropy ceramics discovery

In the format provided by the authors and unedited

Disordered enthalpy-entropy descriptor for high-entropy ceramics discovery

Simon Divilov,[1,2] Hagen Eckert,[1,2] David Hicks,[1,2] Corey Oses,[1,2] Cormac Toher,[3,2] Rico Friedrich,[4,5,2] Marco Esters,[1,2] Michael J. Mehl,[1,2] Adam C. Zettel,[1,2] Yoav Lederer,[6,2] Eva Zurek,[7] Jon-Paul Maria,[8] Donald W. Brenner,[9] Xiomara Campilongo,[2] Suzana Filipović,[10,11] William G. Fahrenholtz,[10] Caillin J. Ryan,[12] Christopher M. DeSalle,[12] Ryan J. Crealese,[12] Douglas E. Wolfe,[12] Arrigo Calzolari,[1,2,13] and Stefano Curtarolo[1,2,*]

[1] *Department of Mechanical Engineering and Materials Science, Duke University, Durham, NC 27708, USA*
[2] *Center for Autonomous Materials Design, Duke University, Durham, NC 27708, USA*
[3] *Department of Materials Science and Engineering and Department of Chemistry and Biochemistry, University of Texas at Dallas, Richardson, TX 75080, USA*
[4] *Institute of Ion Beam Physics and Materials Research, Helmholtz-Zentrum Dresden-Rossendorf, 01328 Dresden, Germany*
[5] *Theoretische Chemie, Technische Universität Dresden, 01062 Dresden, Germany*
[6] *Department of Physics, NRCN, P.O. Box 9001, Beer-Sheva 84190, Israel*
[7] *Department of Chemistry, State University of New York at Buffalo, Buffalo, NY 14260, USA*
[8] *Department of Materials Science and Engineering, The Pennsylvania State University, University Park, PA 16802, USA*
[9] *Department of Materials Science and Engineering, North Carolina State University, Raleigh, NC 27695, USA*
[10] *Department of Materials Science and Engineering, Missouri University of Science and Technology, Rolla, MO 65409, USA*
[11] *Institute of Technical Sciences of the Serbian Academy of Sciences and Arts, 11000 Belgrade, Serbia*
[12] *Applied Research Laboratory, The Pennsylvania State University, University Park, PA 16802, USA*
[13] *CNR-NANO Research Center S3, 41125 Modena, Italy*

(Dated: October 19, 2023)

* stefano@duke.edu

Supplementary Table 1. Synthesizability data and predictions for high-entropy carbides

Table I presents synthesizability data for all carbides calculated in the aflow.org repository. The dataset includes DEED, compensation temperature Θ, ensemble enthalpy cost $\langle \Delta H_{\mathrm{hull}} \rangle$, ensemble entropy gain $1/\sigma$ (EFA) and VEC. In addition, the single- and multi-phase classifications (●/✖) are reported from the literature or predicted (when no experimental data is available), based on the thresholds observed in Figure 2 of the main text.

Table I. **Stability data for high-entropy carbides.** The carbide compositions (AFLOW prototype AB_cF8_225_a_b) [1–3] are sorted by DEED. DEED and $1/\sigma$ (EFA) are in units of $(\mathrm{eV/atom})^{-1}$, Θ in Kelvin, $\langle \Delta H_{\mathrm{hull}} \rangle$ in (meV/atom), and VEC in (e/cell). Experimental results showing single- and multi-phase forming systems are designated with ● and ✖, respectively, while mixed reports are designated with ■. The **valid.** label refers to results obtained in this work.

**carbides**													
**composition**	**DEED**	Θ	$\langle \Delta H_{\mathrm{hull}} \rangle$	$1/\sigma$	**VEC**	**exp./pr.**	**composition**	**DEED**	Θ	$\langle \Delta H_{\mathrm{hull}} \rangle$	$1/\sigma$	**VEC**	**exp./pr.**
**4-metal systems**							(HfNbTiVW)C	23	514	115	59	8.80	●
(HfNbTaZr)C	65	180	22	92	8.50	● [4]	(MnNbTaVW)C	22	516	205	103	9.60	●
(HfTaTiZr)C	44	263	43	83	8.25	● [4]	(CrMoNbTiV)C	22	521	172	85	9.20	●
(MoNbTaTi)C	40	292	78	122	9.00	●	(CrNbTaTiW)C	22	521	159	79	9.20	●
**5-metal systems**							(AlHfTaTiZr)C	22	522	207	102	8.00	●
(HfNbTaTiZr)C	53	220	36	99	8.40	● [5–14]	(MnNbTaTiV)C	22	523	156	77	9.20	●
(HfScTaTiZr)C	37	311	39	55	8.00	● [15]	(CrHfNbTaTi)C	22	525	129	63	8.80	●
(HfNbTaTiV)C	37	316	77	103	8.60	● [13, 14]	(MnTaTiVW)C	22	530	193	93	9.40	●
(HfMoNbTaTi)C	32	362	81	83	8.80	● [16]	(HfNbTaVW)C	22	534	135	64	9.00	●
(MoNbTaTiV)C	31	369	101	100	9.00	● [16]	(AlHfNbTaZr)C	22	535	167	79	8.20	●
(NbTaTiVZr)C	30	388	89	80	8.60	● [14]	(CrMoNbTaW)C	22	535	214	101	9.60	● [17]
(HfMoNbTaZr)C	28	408	86	70	8.80	●	(HfMoNbTaW)C	22	536	150	70	9.20	●
(HfNbTaTiW)C	28	411	82	66	8.80	● [13]	(CrMoNbVW)C	22	537	229	107	9.60	● [17]
(MoNbTaTiZr)C	28	415	92	72	8.80	●	(HfTaTiVW)C	21	540	121	56	8.80	●
(HfNbTaVZr)C	27	436	93	65	8.60	● [14]	(MoNbTaVZr)C	21	553	135	59	9.00	●
(HfMoNbTiZr)C	27	437	93	65	8.60	●	(CrTaTiVW)C	21	558	172	75	9.20	●
(HfTaTiVZr)C	26	439	104	72	8.40	● [14]	(MoNbTiVW)C	21	558	156	67	9.20	●
(HfNbTiVZr)C	26	440	102	71	8.40	● [14, 18]	(CrMoTaVW)C	21	560	223	96	9.60	● [17]
(MoNbTaVW)C	26	443	171	117	9.40	● [13]	(MoNbTiVZr)C	21	566	136	57	8.80	●
(NbTaTiVW)C	26	446	111	75	9.00	● [13]	(MoTaTiVW)C	20	570	153	63	9.20	●
(MnMoNbTaV)C	26	448	191	128	9.60	●	(MoTaTiVZr)C	20	573	139	57	8.80	●
(HfMoTaTiZr)C	25	458	95	61	8.60	● [16]	(AlMoNbTaV)C	20	575	204	83	8.80	●
(HfNbTaWZr)C	25	460	92	59	8.80	●	(HfTaTiYZr)C	20	583	110	44	8.00	●
(CrMoNbTaTi)C	25	460	148	94	9.20	●	(NbTiVWZr)C	20	584	128	51	8.80	●
(NbTaTiWZr)C	25	460	92	58	8.80	● [19]	(HfMoNbTiW)C	20	586	133	52	9.00	●
(HfNbTiWZr)C	25	463	84	53	8.60	●	(CrNbTaTiZr)C	20	589	140	54	8.80	●
(CrNbTaTiV)C	25	465	137	85	9.00	●	(MoNbTaWZr)C	20	589	159	62	9.20	●
(CrMoNbTaV)C	25	467	174	108	9.40	●	(MnNbTaTiW)C	20	591	186	72	9.40	●
(HfMoNbTaV)C	24	479	123	72	9.00	●	(CrMoNbTiW)C	20	594	203	77	9.40	●
(HfMoNbTiV)C	24	488	122	69	8.80	●	(HfMoTaTiW)C	19	603	138	51	9.00	●
(MoNbTaTiW)C	24	489	134	75	9.20	●	(CrMoTiVW)C	19	605	208	76	9.40	● [17]
(AlHfNbTaV)C	24	493	185	103	8.40	●	(TaTiVWZr)C	19	607	134	49	8.80	✖
(HfTaTiWZr)C	23	495	90	49	8.60	● [13]	(NbTaVWZr)C	19	610	147	53	9.00	✖
(HfMoTaTiV)C	23	497	124	68	8.80	●	(MnMoNbVW)C	19	612	248	89	9.80	✖
(CrNbTaVW)C	23	497	185	101	9.40	●	(CrMoTaTiW)C	19	618	199	70	9.40	✖
(MnMoNbTaTi)C	23	505	170	90	9.40	●	(HfMoNbVZr)C	19	623	144	50	8.80	✖
(MnNbTiVW)C	23	505	188	99	9.40	●	(HfScTiYZr)C	19	623	195	67	7.60	✖
(CrMoTaTiV)C	23	506	166	87	9.20	●	(CrHfNbTiW)C	19	626	166	57	9.00	✖
(CrNbTiVW)C	23	510	166	86	9.20	●	(CrHfTaTiV)C	18	629	166	56	8.80	✖

carbides

composition	DEED	Θ	$\langle \Delta H_{\mathrm{hull}} \rangle$	$1/\sigma$	VEC	exp./pr.	composition	DEED	Θ	$\langle \Delta H_{\mathrm{hull}} \rangle$	$1/\sigma$	VEC	exp./pr.
(HfMoTaVZr)C	18	633	146	49	8.80	✖	(CrNbTaWZr)C	16	747	186	45	9.20	✖
(HfMoTiVZr)C	18	635	151	50	8.60	✖	(MnMoNbTaZr)C	15	750	201	48	9.40	✖
(MnMoTaVW)C	18	635	242	81	9.80	✖	(IrNbTiVW)C	15	750	404	97	9.60	✖
(HfTiVWZr)C	18	636	139	46	8.60	✖	(MnTaTiWZr)C	15	751	201	48	9.20	✖
(MoNbTiWZr)C	18	642	144	47	9.00	✖	(MoTaVWZr)C	15	752	195	46	9.20	✖
(AlHfMoNbTi)C	18	645	213	69	8.40	✖	(CrHfMoTiV)C	15	752	199	47	9.00	✖
(CrHfTaTiZr)C	18	645	159	51	8.60	✖	(CrNbTiVZr)C	15	753	181	43	8.80	✖
(CrHfMoNbTi)C	18	646	174	56	9.00	✖	(AlHfTiVZr)C	15	753	269	64	8.00	✖
(CrHfTaTiW)C	18	647	170	55	9.00	✖	(IrTaTiVW)C	15	753	415	99	9.60	✖
(HfMoNbWZr)C	18	647	146	47	9.00	✖	(AlCrTaVW)C	15	753	248	59	9.00	✖
(CrHfMoTaTi)C	18	647	174	56	9.00	✖	(IrMoTaTiV)C	15	756	409	96	9.60	✖
(CrHfNbTiV)C	18	649	163	52	8.80	✖	(CrHfTiWZr)C	15	765	190	44	8.80	✖
(HfNbVWZr)C	18	651	142	45	8.80	✖	(CrHfNbVW)C	15	766	204	47	9.20	✖
(CrHfMoNbTa)C	18	653	167	53	9.20	✖	(CrHfMoNbV)C	15	766	206	47	9.20	✖
(HfMnTaTiW)C	18	653	188	59	9.20	✖	(CrHfTaVW)C	15	767	208	48	9.20	✖
(HfMnMoTaTi)C	18	654	191	60	9.20	✖	(MnMoNbTiZr)C	15	767	204	47	9.20	✖
(MnMoNbTiW)C	18	655	219	69	9.60	✖	(MnNbTiWZr)C	15	768	198	45	9.20	✖
(MoTaTiWZr)C	17	664	150	46	9.00	✖	(AlHfMoTiZr)C	15	771	250	57	8.20	✖
(MnMoTaTiW)C	17	668	218	66	9.60	✖	(CrHfMoTaW)C	15	772	224	51	9.40	✖
(HfMoNbVW)C	17	668	184	56	9.20	✖	(AlTaTiWZr)C	15	777	206	46	8.40	✖
(HfMnMoNbTa)C	17	673	189	56	9.40	✖	(IrNbTaTiW)C	15	778	392	87	9.60	✖
(HfMoTaVW)C	17	673	183	54	9.20	✖	(HfMnMoTiW)C	15	780	230	51	9.40	✖
(CrHfNbTiZr)C	17	674	157	47	8.60	✖	(MoTiVWZr)C	15	780	179	40	9.00	✖
(AlHfMoTaTi)C	17	675	211	62	8.40	✖	(HfMnNbTaW)C	15	780	210	46	9.40	✖
(HfTaVWZr)C	17	679	148	43	8.80	✖	(HfMnTiVW)C	15	782	208	46	9.20	✖
(MnMoTiVW)C	17	680	227	66	9.60	✖	(CrHfMoNbW)C	15	784	226	50	9.40	✖
(HfMoTaWZr)C	17	681	152	44	9.00	✖ [13]	(IrMoNbTiV)C	15	785	406	89	9.60	✖
(CrHfNbTaZr)C	17	682	144	42	8.80	✖	(IrMoTiVW)C	15	793	451	97	9.80	✖
(MnMoTaTiV)C	17	682	194	56	9.40	✖	(CrNbTaVZr)C	15	797	182	38	9.00	✖
(HfMnNbTiW)C	17	683	184	53	9.20	✖	(HfMnNbTaTi)C	14	801	155	32	9.00	✖
(AlMnNbTiV)C	17	688	275	78	8.80	✖	(AlMnMoTaTi)C	14	807	290	60	9.00	✖
(IrMoNbTaV)C	17	689	417	118	9.80	✖	(IrNbTaTiV)C	14	807	363	75	9.40	✖
(IrNbTaVW)C	17	693	434	122	9.80	✖	(AlNbVWZr)C	14	815	246	50	8.60	✖
(MnMoNbTaW)C	17	694	234	65	9.80	✖	(IrMoTaTiW)C	14	816	443	90	9.80	✖
(AlCrHfNbTa)C	17	695	234	65	8.60	✖	(CrHfMoTiZr)C	14	817	203	41	8.80	✖
(HfMoTiVW)C	17	701	164	45	9.00	✖	(HfMnMoTaW)C	14	817	254	51	9.60	✖
(HfMoTiWZr)C	17	702	141	39	8.80	✖ [13]	(CrHfMoTiW)C	14	819	210	42	9.20	✖ [17]
(CrHfNbTaW)C	16	704	178	49	9.20	✖	(CrTiVWZr)C	14	821	207	41	9.00	✖
(CrHfNbTaV)C	16	705	168	46	9.00	✖	(MoNbSiTaTi)C	14	822	327	65	8.80	✖
(CrNbTiWZr)C	16	707	178	48	9.00	✖	(CrHfNbWZr)C	14	824	191	38	9.00	✖
(IrMoNbTaTi)C	16	714	380	100	9.60	✖	(AlCrHfMoTa)C	14	826	287	57	8.80	✖
(CrHfTiVW)C	16	714	188	50	9.00	✖	(CrHfMoTaZr)C	14	829	196	38	9.00	✖
(MnMoNbTiV)C	16	715	201	53	9.40	✖	(AlMnMoNbTi)C	14	832	299	58	9.00	✖
(HfMnMoNbTi)C	16	721	193	50	9.20	✖	(CrHfMoNbZr)C	14	833	194	38	9.00	✖
(CrMoNbTaZr)C	16	724	178	46	9.20	✖	(HfMnMoTiV)C	14	835	220	42	9.20	✖
(AlMnNbVW)C	16	725	307	79	9.20	✖	(SiTaTiVW)C	14	839	319	61	8.80	✖
(CrTaTiWZr)C	16	731	183	46	9.00	✖	(MnTiVWZr)C	14	839	225	43	9.20	✖
(MoNbVWZr)C	16	732	188	47	9.20	✖	(HfMnMoNbW)C	14	841	254	48	9.60	✖
(CrHfMoTaV)C	16	737	200	50	9.20	✖	(IrMoNbVW)C	14	842	478	91	10.00	✖
(CrMoNbTiZr)C	16	738	187	46	9.00	✖	(AlCrMnTaV)C	14	845	311	59	9.20	✖
(AlCrHfNbTi)C	16	738	261	64	8.40	✖	(CrHfTiVZr)C	14	846	203	38	8.60	✖
(CrTaTiVZr)C	16	739	182	45	8.80	✖	(IrMoTaVW)C	14	846	483	91	10.00	✖
(CrMoTaTiZr)C	16	740	190	47	9.00	✖	(AlMnMoTaV)C	14	846	301	57	9.20	✖
(AlNbTiWZr)C	16	747	206	50	8.40	✖	(HfMoVWZr)C	14	846	195	37	9.00	✖ [13]

carbides													
**composition**	**DEED**	Θ	$\langle \Delta H_{\text{hull}} \rangle$	$1/\sigma$	**VEC**	**exp./pr.**	**composition**	**DEED**	Θ	$\langle \Delta H_{\text{hull}} \rangle$	$1/\sigma$	**VEC**	**exp./pr.**
(CrHfTaWZr)C	14	851	197	37	9.00	✖ [17]	(CrHfVWZr)C	12	968	234	34	9.00	✖
(CrMoTaVZr)C	14	852	216	40	9.20	✖	(MnTaVWZr)C	12	971	256	37	9.40	✖
(IrMoNbTiW)C	14	854	437	81	9.80	✖	(AlCrHfTiZr)C	12	979	319	45	8.20	✖
(HfMnMoVW)C	14	857	269	49	9.60	✖	(HfMnMoTaZr)C	12	980	217	30	9.20	✖
(CrMoNbVZr)C	13	860	213	39	9.20	✖	(IrMnNbTaV)C	12	981	446	62	10.00	✖
(AlCrHfTaZr)C	13	861	287	52	8.40	✖	(IrMnMoTiV)C	12	984	466	65	10.00	✖
(CrMoTaWZr)C	13	863	236	43	9.40	✖	(IrMnMoNbW)C	12	984	533	74	10.40	✖
(HfMnTaVW)C	13	863	239	43	9.40	✖	(CrNbSiTiW)C	12	986	367	51	9.00	✖
(NbSiTiVW)C	13	865	313	56	8.80	✖	(CrNbSiTaW)C	12	988	390	54	9.20	✖
(CrMoNbWZr)C	13	865	229	41	9.40	✖	(MnNbVWZr)C	12	992	253	35	9.40	✖
(MnMoTiWZr)C	13	867	247	44	9.40	✖	(AlHfMnTaW)C	12	998	314	43	9.00	✖
(CrMoTiVZr)C	13	868	218	39	9.00	✖	(IrMnMoVW)C	12	1,007	544	72	10.40	✖
(AlCrMoTaW)C	13	868	298	53	9.20	✖	(MnTaTiVZr)C	12	1,009	209	28	9.00	✖
(MnNbTaTiZr)C	13	869	166	30	9.00	✖	(MnMoNbVZr)C	11	1,010	244	32	9.40	✖
(MnNbTaWZr)C	13	870	221	39	9.40	✖	(CrNbSiTiV)C	11	1,010	345	46	8.80	✖
(CrHfMoVW)C	13	871	249	44	9.40	✖	(IrMnTaTiW)C	11	1,013	466	61	10.00	✖
(CrNbVWZr)C	13	871	219	39	9.20	✖	(IrMnMoTaW)C	11	1,014	537	70	10.40	✖
(AlCrHfMoTi)C	13	872	295	52	8.60	✖	(HfIrMoNbTa)C	11	1,018	383	50	9.60	✖
(CrTaVWZr)C	13	878	224	39	9.20	✖	(MnNbTaVZr)C	11	1,020	209	27	9.20	✖
(MnMoTaWZr)C	13	892	266	45	9.60	✖	(CrHfMoVZr)C	11	1,022	243	31	9.00	✖
(AlMnNbTaTi)C	13	893	261	44	8.80	✖	(CrHfMoWZr)C	11	1,023	239	31	9.20	✖
(HfMnMoTaV)C	13	897	230	39	9.40	✖	(IrMnMoTiW)C	11	1,023	511	66	10.20	✖
(MnMoNbWZr)C	13	897	257	43	9.60	✖	(CrNbSiVW)C	11	1,023	395	51	9.20	✖
(IrMnNbVW)C	13	899	499	83	10.20	✖	(CrMoSiTaTi)C	11	1,031	387	49	9.00	✖
(CrMoTiWZr)C	13	901	225	37	9.20	✖ [17]	(HfMnTaWZr)C	11	1,033	217	27	9.20	✖
(CrHfTaVZr)C	13	902	201	33	8.80	✖	(HfIrNbTaTi)C	11	1,036	323	40	9.20	✖
(IrMnMoNbV)C	13	902	489	81	10.20	✖	(HfMnTiWZr)C	11	1,044	211	26	9.00	✖
(AlMnMoNbV)C	13	903	315	52	9.20	✖	(AlCrHfMoZr)C	11	1,045	334	41	8.60	✖
(IrMnTaVW)C	13	903	502	83	10.20	✖	(MnNbTiVZr)C	11	1,052	209	25	9.00	✖
(CrHfNbVZr)C	13	911	200	33	8.80	✖	(HfMnNbWZr)C	11	1,053	212	26	9.20	✖
(IrMnNbTaW)C	13	913	484	78	10.20	✖	(IrMnTaTiV)C	11	1,054	435	53	9.80	✖
(IrMoNbTaW)C	13	914	460	74	10.00	✖	(CrMoNbSiTa)C	11	1,057	399	48	9.20	✖
(HfMnNbVW)C	13	914	240	39	9.40	✖	(CrMoNbSiW)C	11	1,058	448	54	9.40	✖
(AlCrNbWZr)C	13	918	293	47	8.80	✖	(AlIrMoTaW)C	11	1,060	642	77	9.60	✖
(MoNbSiVW)C	13	919	399	64	9.20	✖	(IrMnNbTiW)C	11	1,080	456	53	10.00	✖
(IrMnMoNbTa)C	13	919	468	75	10.20	✖	(HfMnNbTaZr)C	11	1,080	184	21	9.00	✖
(HfMnMoNbV)C	12	931	236	37	9.40	✖	(MoSiTaTiZr)C	11	1,081	362	42	8.60	✖
(MnMoTiVZr)C	12	934	238	37	9.20	✖	(HfSiTaVW)C	11	1,083	360	41	8.80	✖
(MnMoVWZr)C	12	935	274	42	9.60	✖	(HfNbSiVW)C	11	1,085	354	40	8.80	✖
(MoNbSiTiV)C	12	936	335	52	8.80	✖	(HfIrNbTaW)C	11	1,088	400	46	9.60	✖
(HfMnTaTiV)C	12	937	193	30	9.00	✖	(HfIrMoTaTi)C	11	1,091	383	43	9.40	✖
(MnMoTaVZr)C	12	943	246	37	9.40	✖	(AlSiTaTiW)C	11	1,091	373	42	8.40	✖
(IrMnTiVW)C	12	943	461	70	10.00	✖	(HfIrNbTiW)C	11	1,092	371	42	9.40	✖
(MoSiTaTiW)C	12	943	368	56	9.00	✖	(MoNbSiTaZr)C	11	1,093	362	41	8.80	✖
(HfMnNbTiV)C	12	944	191	29	9.00	✖	(HfMnMoWZr)C	11	1,095	267	30	9.40	✖
(CrNbSiTaTi)C	12	948	347	52	8.80	✖	(HfIrMoNbTi)C	11	1,097	376	42	9.40	✖
(AlCrHfMoV)C	12	950	318	47	8.80	✖	(HfIrMoTaV)C	11	1,097	424	47	9.60	✖
(MnMoTaTiZr)C	12	953	215	32	9.20	✖	(CrMnSiTaV)C	11	1,098	427	48	9.40	✖
(IrMnMoTaV)C	12	955	491	72	10.20	✖	(HfNbSiTiV)C	11	1,098	327	36	8.40	✖
(HfMnNbTaV)C	12	956	197	29	9.20	✖	(HfIrTaVW)C	11	1,099	435	49	9.60	✖
(HfNbSiTaTi)C	12	961	299	44	8.40	✖	(AlHfMnMoTi)C	11	1,099	335	37	8.80	✖
(HfMnMoNbZr)C	12	963	215	31	9.20	✖	(HfIrTaTiW)C	11	1,100	381	42	9.40	✖
(AlCrMnNbTi)C	12	965	324	47	9.00	✖	(AlCrMnNbTa)C	11	1,104	321	35	9.20	✖
(CrMoVWZr)C	12	966	257	37	9.40	✖	(AlIrMoNbTa)C	10	1,106	580	64	9.40	✖

carbides													
**composition**	**DEED**	Θ	$\langle\Delta H_{\mathrm{hull}}\rangle$	$1/\sigma$	**VEC**	**exp./pr.**	**composition**	**DEED**	Θ	$\langle\Delta H_{\mathrm{hull}}\rangle$	$1/\sigma$	**VEC**	**exp./pr.**
(HfIrTaTiV)C	10	1,111	364	40	9.20	✖	(IrMoTaWZr)C	9	1,225	454	41	9.80	✖
(HfMnTaTiZr)C	10	1,112	198	22	8.80	✖	(HfMnTiVZr)C	9	1,226	239	21	8.80	✖
(MnSiTiVW)C	10	1,114	399	43	9.20	✖	(AlMoSiTaTi)C	9	1,226	421	38	8.40	✖
(NbSiTaWZr)C	10	1,114	347	38	8.80	✖	(HfIrNbTaZr)C	9	1,227	319	29	9.20	✖
(HfMnTaVZr)C	10	1,118	223	24	9.00	✖	(IrMoTaVZr)C	9	1,227	429	38	9.60	✖
(IrMoNbTaZr)C	10	1,119	385	41	9.60	✖	(IrMoNbWZr)C	9	1,234	441	39	9.80	✖
(AlIrMoVW)C	10	1,119	665	71	9.60	✖	(AlHfNbSiW)C	9	1,236	415	37	8.40	✖
(MnMoNbSiW)C	10	1,119	473	51	9.60	✖	(IrNbTiVZr)C	9	1,239	368	32	9.20	✖
(HfIrNbVW)C	10	1,122	425	45	9.60	✖	(MnMoSiVW)C	9	1,239	465	41	9.60	✖
(IrNbTaTiZr)C	10	1,122	328	35	9.20	✖	(MoNbSiVZr)C	9	1,240	390	34	8.80	✖
(HfIrNbTaV)C	10	1,125	377	40	9.40	✖	(CrHfMoSiTi)C	9	1,242	409	36	8.80	✖
(HfIrMoNbV)C	10	1,125	421	45	9.60	✖	(AlCrNbSiV)C	9	1,242	424	37	8.60	✖
(IrMnNbTiV)C	10	1,126	428	45	9.80	✖	(IrNbTaVZr)C	9	1,244	384	33	9.40	✖
(HfIrMoNbW)C	10	1,129	444	47	9.80	✖	(IrMoNbVZr)C	9	1,246	420	36	9.60	✖
(HfIrNbTiV)C	10	1,130	357	38	9.20	✖	(CrMnNbSiTa)C	9	1,246	439	38	9.40	✖
(HfMnVWZr)C	10	1,133	261	27	9.20	✖	(HfMnMoVZr)C	9	1,247	273	24	9.20	✖
(HfMoNbSiV)C	10	1,133	375	39	8.80	✖	(HfMoSiTiZr)C	9	1,249	376	32	8.40	✖
(MnMoSiTaW)C	10	1,137	468	49	9.60	✖	(IrTaVWZr)C	9	1,250	443	38	9.60	✖
(AlMnTaVZr)C	10	1,139	337	35	8.80	✖	(IrNbVWZr)C	9	1,258	432	37	9.60	✖
(HfMnNbTiZr)C	10	1,140	201	21	8.80	✖	(MoSiTiWZr)C	9	1,258	384	33	8.80	✖
(SiTaTiVZr)C	10	1,142	345	36	8.40	✖	(MnMoSiTiW)C	9	1,264	445	38	9.40	✖
(SiTiVWZr)C	10	1,146	359	37	8.60	✖	(CrNbSiTaZr)C	9	1,265	394	33	8.80	✖
(AlHfSiTaW)C	10	1,152	407	41	8.40	✖	(IrMnMoNbTi)C	9	1,266	468	39	10.00	✖
(HfSiTaTiZr)C	10	1,156	331	33	8.20	✖	(CrSiTiWZr)C	9	1,272	408	34	8.80	✖
(HfIrMoTaW)C	10	1,156	454	46	9.80	✖	(IrMnNbTaTi)C	9	1,274	421	35	9.80	✖
(HfMnMoTiZr)C	10	1,156	236	24	9.00	✖	(CrMnMoSiTi)C	9	1,277	447	37	9.40	✖
(AlNbSiTaV)C	10	1,159	366	37	8.40	✖	(AlCrMoSiTi)C	9	1,281	451	37	8.60	✖
(MnMoNbSiTi)C	10	1,161	411	41	9.20	✖	(IrMoTiWZr)C	9	1,283	429	35	9.60	✖
(HfIrTaTiZr)C	10	1,167	316	31	9.00	✖	(CrMnMoSiW)C	9	1,285	505	41	9.80	✖
(AlMnMoTaZr)C	10	1,170	339	33	9.00	✖	(AlMnSiVW)C	9	1,287	479	39	9.00	✖
(HfIrMoTiW)C	10	1,173	425	42	9.60	✖	(CrHfMoSiV)C	9	1,301	432	34	9.00	✖
(HfSiTiWZr)C	10	1,178	349	34	8.40	✖	(AlIrMnMoV)C	9	1,306	687	54	9.80	✖
(AlIrTaVW)C	10	1,180	615	59	9.40	✖	(HfIrMnMoTa)C	9	1,309	473	37	10.00	✖
(NbSiTiVZr)C	10	1,189	345	33	8.40	✖	(IrTiVWZr)C	9	1,313	411	32	9.40	✖
(CrHfSiTaV)C	10	1,192	398	38	8.80	✖	(AlHfMoNbSi)C	9	1,314	456	36	8.40	✖
(HfIrTiVW)C	10	1,192	404	38	9.40	✖	(HfIrMnMoW)C	9	1,320	528	41	10.20	✖
(IrMoTaTiZr)C	10	1,197	389	37	9.40	✖	(IrMoTiVZr)C	9	1,321	417	32	9.40	✖
(CrMnSiTiV)C	10	1,198	395	37	9.20	✖	(HfIrTiVZr)C	9	1,330	364	28	9.00	✖
(HfIrNbTiZr)C	10	1,199	313	29	9.00	✖	(AlSiVWZr)C	9	1,331	457	35	8.40	✖
(AlMnMoNbZr)C	10	1,199	339	32	9.00	✖	(AlIrTaTiV)C	9	1,332	562	43	9.00	✖
(IrNbTiWZr)C	10	1,200	377	35	9.40	✖	(MnNbSiTaV)C	9	1,332	403	31	9.20	✖
(AlSiTaTiV)C	10	1,200	375	35	8.20	✖	(HfMnSiTaW)C	9	1,332	454	34	9.20	✖
(IrMoNbTiZr)C	10	1,201	382	36	9.40	✖	(AlNbSiTaZr)C	9	1,334	407	31	8.20	✖
(HfMnNbVZr)C	10	1,201	226	21	9.00	✖	(AlHfMoSiTa)C	9	1,334	456	34	8.40	✖
(IrNbTaWZr)C	10	1,202	404	38	9.60	✖	(HfIrTaVZr)C	9	1,335	375	28	9.20	✖
(HfMoSiTaW)C	10	1,205	397	37	9.00	✖	(AlMoNbSiZr)C	9	1,337	470	35	8.40	✖
(SiTaVWZr)C	10	1,205	375	35	8.80	✖	(CrMnSiVW)C	9	1,338	460	35	9.60	✖
(HfIrMoTiV)C	10	1,205	409	38	9.40	✖	(IrMoVWZr)C	9	1,341	476	36	9.80	✖
(CrHfSiTaTi)C	10	1,206	382	35	8.60	✖	(HfIrTiWZr)C	9	1,341	362	27	9.20	✖
(HfIrMoVW)C	10	1,212	474	43	9.80	✖	(HfIrTaWZr)C	9	1,342	383	29	9.40	✖
(IrTaTiWZr)C	10	1,213	387	35	9.40	✖	(HfIrNbWZr)C	9	1,342	373	28	9.40	✖
(IrMnMoTaTi)C	10	1,214	470	43	10.00	✖	(HfIrMoTaZr)C	9	1,343	382	28	9.40	✖
(AlHfSiTiW)C	10	1,214	415	38	8.20	✖	(CrSiTaVZr)C	9	1,344	419	31	8.80	✖
(IrTaTiVZr)C	10	1,221	375	34	9.20	✖	(HfIrMoNbZr)C	9	1,346	374	28	9.40	✖

carbides													
**composition**	**DEED**	Θ	$\langle \Delta H_{\mathrm{hull}} \rangle$	$1/\sigma$	**VEC**	**exp./pr.**	**composition**	**DEED**	Θ	$\langle \Delta H_{\mathrm{hull}} \rangle$	$1/\sigma$	**VEC**	**exp./pr.**
(AlCrMnTaZr)C	9	1,346	383	28	9.00	✖	(AlHfMnSiTi)C	8	1,530	534	31	8.40	✖
(AlIrMoTaTi)C	9	1,346	579	43	9.20	✖	(CrHfMoSiZr)C	8	1,532	454	26	8.80	✖
(CrNbSiTiZr)C	9	1,351	398	29	8.60	✖	(HfIrMnNbV)C	8	1,534	462	26	9.80	✖
(AlIrMoTiV)C	9	1,355	608	45	9.20	✖	(IrMnSiTiW)C	8	1,536	672	38	9.80	✖
(AlHfMnTiZr)C	9	1,359	371	27	8.40	✖	(HfIrMnNbTa)C	8	1,536	431	25	9.80	✖
(AlHfNbSiV)C	9	1,359	429	31	8.20	✖	(MnMoSiTaZr)C	8	1,540	465	26	9.20	✖
(CrMnSiTaTi)C	9	1,361	433	31	9.20	✖	(HfIrMnNbTi)C	8	1,544	418	24	9.60	✖
(AlCrIrTaV)C	9	1,362	627	46	9.40	✖	(IrMnSiTaTi)C	7	1,549	640	36	9.60	✖
(CrSiVWZr)C	9	1,362	433	31	9.00	✖	(IrMnMoSiTa)C	7	1,553	689	38	10.00	✖
(HfIrNbVZr)C	9	1,362	370	27	9.20	✖	(MnSiVWZr)C	7	1,567	480	26	9.20	✖
(AlCrHfSiTi)C	9	1,365	476	34	8.20	✖	(AlCrIrMnNb)C	7	1,568	695	38	9.80	✖
(HfIrMoTiZr)C	8	1,366	374	27	9.20	✖	(AlCrMoSiZr)C	7	1,572	534	29	8.60	✖
(AlCrMnNbZr)C	8	1,371	387	28	9.00	✖	(IrMnSiTaW)C	7	1,574	705	38	10.00	✖
(AlIrNbTiW)C	8	1,373	574	41	9.20	✖	(MnNbSiTaZr)C	7	1,579	435	23	9.00	✖
(AlCrIrMoNb)C	8	1,374	658	47	9.60	✖	(IrMnNbWZr)C	7	1,580	486	26	10.00	✖
(AlIrMnTiV)C	8	1,376	630	45	9.40	✖	(IrNbSiVW)C	7	1,580	665	36	9.60	✖
(AlCrHfSiW)C	8	1,396	480	33	8.60	✖	(IrMnVWZr)C	7	1,582	510	27	10.00	✖
(AlIrMnMoW)C	8	1,400	724	50	10.00	✖	(IrMoSiTaV)C	7	1,584	645	35	9.60	✖
(AlHfMoSiW)C	8	1,402	473	32	8.60	✖	(CrIrNbSiTi)C	7	1,586	620	33	9.40	✖
(AlCrIrTaTi)C	8	1,407	610	41	9.20	✖	(IrMnMoVZr)C	7	1,591	504	27	10.00	✖
(HfIrMnTaTi)C	8	1,414	415	28	9.60	✖	(IrMnTaWZr)C	7	1,592	498	26	10.00	✖
(AlMnNbSiTa)C	8	1,414	469	32	8.80	✖	(HfIrMnMoTi)C	7	1,594	477	25	9.80	✖
(AlCrMnSiW)C	8	1,420	521	35	9.20	✖	(IrMnTiVZr)C	7	1,598	447	24	9.60	✖
(AlCrMnMoZr)C	8	1,421	415	28	9.20	✖	(CrMnSiWZr)C	7	1,599	523	28	9.40	✖
(MnMoNbSiZr)C	8	1,422	457	30	9.20	✖	(MnSiTiVZr)C	7	1,605	447	23	8.80	✖
(AlHfIrTaTi)C	8	1,426	541	36	8.80	✖	(AlIrNbWZr)C	7	1,612	595	31	9.20	✖
(CrHfSiWZr)C	8	1,432	434	28	8.80	✖	(AlIrMoNbZr)C	7	1,612	592	31	9.20	✖
(HfIrMoWZr)C	8	1,435	430	28	9.60	✖	(IrMnTiWZr)C	7	1,614	470	24	9.80	✖
(AlHfMnTaZr)C	8	1,438	351	23	8.60	✖	(AlIrNbVZr)C	7	1,618	589	30	9.00	✖
(AlHfSiTaZr)C	8	1,441	442	29	8.00	✖	(IrMnTaVZr)C	7	1,620	478	25	9.80	✖
(IrMnMoTaZr)C	8	1,443	477	31	10.00	✖	(IrMnMoNbZr)C	7	1,628	479	24	10.00	✖
(HfIrMnMoV)C	8	1,444	500	32	10.00	✖	(IrMoNbSiW)C	7	1,629	688	35	9.80	✖
(HfIrMnVW)C	8	1,453	504	32	10.00	✖	(IrMnTaTiZr)C	7	1,632	434	22	9.60	✖
(HfMnMoSiTa)C	8	1,454	451	29	9.20	✖	(IrMnNbTiZr)C	7	1,634	430	22	9.60	✖
(HfIrVWZr)C	8	1,455	419	27	9.40	✖	(IrMnNbTaZr)C	7	1,639	436	22	9.80	✖
(HfMnSiVW)C	8	1,456	461	29	9.20	✖	(IrMnNbVZr)C	7	1,641	472	24	9.80	✖
(HfIrMnTiV)C	8	1,460	437	28	9.60	✖	(AlCrHfIrTa)C	7	1,649	626	31	9.20	✖
(HfIrMnTaW)C	8	1,464	491	31	10.00	✖	(CrIrMnSiTi)C	7	1,669	667	32	9.80	✖
(AlCrIrMoTi)C	8	1,467	654	41	9.40	✖	(CrIrSiTaW)C	7	1,672	695	33	9.80	✖
(HfIrMoVZr)C	8	1,468	420	26	9.40	✖	(AlMnMoSiZr)C	7	1,686	559	27	8.80	✖
(HfIrMnNbW)C	8	1,469	480	30	10.00	✖	(CrIrMoSiV)C	7	1,686	682	32	9.80	✖
(AlMnMoSiV)C	8	1,483	490	30	9.00	✖	(AlHfIrVW)C	7	1,689	619	29	9.20	✖
(HfIrMnTaV)C	8	1,490	467	28	9.80	✖	(IrMnMoTiZr)C	7	1,691	485	23	9.80	✖
(CrIrSiTiV)C	8	1,491	622	38	9.40	✖	(IrMoSiTaTi)C	7	1,697	620	29	9.40	✖
(AlMnMoSiTi)C	8	1,501	492	29	8.80	✖	(CrMnMoSiZr)C	7	1,712	522	24	9.40	✖
(HfIrMnMoNb)C	8	1,509	479	28	10.00	✖	(HfIrMnTiZr)C	7	1,714	427	20	9.40	✖
(HfIrMnTiW)C	8	1,511	463	27	9.80	✖	(AlHfMnMoSi)C	7	1,719	545	25	8.80	✖
(AlMnMoNbSi)C	8	1,513	506	30	9.00	✖	(AlHfIrVZr)C	7	1,725	604	27	8.80	✖
(AlHfIrNbV)C	8	1,514	578	34	9.00	✖	(AlIrSiTaTi)C	7	1,730	707	32	8.80	✖
(AlHfIrTaW)C	8	1,515	589	35	9.20	✖	(AlCrIrSiTi)C	7	1,736	754	34	9.00	✖
(AlIrTaTiZr)C	8	1,522	551	32	8.80	✖	(AlCrIrTaZr)C	7	1,754	636	28	9.20	✖
(IrMnMoWZr)C	8	1,522	534	31	10.20	✖	(AlCrIrNbZr)C	7	1,761	636	28	9.20	✖
(CrHfSiTiZr)C	8	1,526	423	24	8.40	✖	(AlIrSiVW)C	7	1,768	764	33	9.20	✖
(AlHfMoSiZr)C	8	1,528	501	29	8.20	✖	(CrHfIrNbSi)C	7	1,769	648	28	9.40	✖

carbides													
**composition**	**DEED**	Θ	$\langle \Delta H_{\mathrm{hull}} \rangle$	$1/\sigma$	**VEC**	**exp./pr.**	**composition**	**DEED**	Θ	$\langle \Delta H_{\mathrm{hull}} \rangle$	$1/\sigma$	**VEC**	**exp./pr.**
(AlMnSiVZr)C	7	1,770	562	24	8.60	✖	(IrMoSiVZr)C	6	1,953	672	24	9.40	✖
(HfIrMnWZr)C	7	1,771	468	20	9.80	✖	(AlHfIrSiV)C	6	1,954	752	27	8.80	✖
(AlCrIrSiV)C	7	1,771	780	33	9.20	✖	(IrNbSiWZr)C	6	1,963	658	23	9.40	✖
(AlIrMoSiTa)C	6	1,792	773	32	9.20	✖	(IrSiVWZr)C	6	1,963	680	24	9.40	✖
(AlCrIrSiTa)C	6	1,794	776	32	9.20	✖	(IrMoSiTiZr)C	6	1,972	640	22	9.20	✖
(AlIrSiTaW)C	6	1,802	752	31	9.20	✖	(AlCrIrMnSi)C	6	1,974	859	30	9.60	✖
(CrIrMnSiW)C	6	1,814	737	30	10.20	✖	(IrSiTiWZr)C	6	1,985	632	22	9.20	✖
(AlIrMnSiV)C	6	1,817	816	33	9.40	✖	(AlHfIrMnMo)C	6	1,989	695	24	9.60	✖
(HfIrMoNbSi)C	6	1,829	629	25	9.40	✖	(CrIrMoSiZr)C	6	2,012	703	23	9.60	✖
(HfIrMnVZr)C	6	1,829	475	19	9.60	✖	(AlIrSiTaZr)C	6	2,026	734	24	8.80	✖
(AlIrMoNbSi)C	6	1,829	781	31	9.20	✖	(HfIrSiVZr)C	6	2,026	627	21	9.00	✖
(IrNbSiTiZr)C	6	1,852	588	23	9.00	✖	(AlHfIrMnW)C	6	2,041	679	22	9.60	✖
(HfIrMnMoZr)C	6	1,859	486	19	9.80	✖	(CrIrMnSiZr)C	5	2,128	751	22	9.80	✖
(HfIrMnNbZr)C	6	1,864	434	17	9.60	✖	(AlHfIrSiW)C	5	2,160	755	22	9.00	✖
(CrHfIrMoSi)C	6	1,874	695	27	9.60	✖	(AlHfIrSiZr)C	5	2,163	755	22	8.60	✖
(AlHfIrSiTi)C	6	1,877	727	28	8.60	✖	(AlHfIrMoSi)C	5	2,166	780	22	9.00	✖
(HfIrMnTaZr)C	6	1,878	439	17	9.60	✖	(AlIrMnMoZr)C	5	2,172	702	20	9.60	✖
(HfIrMnMoSi)C	6	1,883	704	27	9.80	✖	(AlCrHfIrSi)C	5	2,178	803	23	9.00	✖
(IrNbSiTaZr)C	6	1,898	597	22	9.20	✖	(AlCrIrMnZr)C	5	2,190	731	21	9.60	✖
(AlHfIrNbSi)C	6	1,926	723	26	8.80	✖	(AlHfIrMnZr)C	5	2,262	674	18	9.20	✖

Supplementary Table 2. Synthesizability data and predictions for high-entropy carbonitrides

Table II presents synthesizability data for all carbonitrides calculated in the aflow.org repository. The dataset includes DEED, compensation temperature Θ, ensemble enthalpy cost $\langle \Delta H_{\mathrm{hull}} \rangle$, ensemble entropy gain $1/\sigma$ (EFA) and VEC. In addition, the single- and multi-phase classifications (●/✖) are reported from the literature or predicted (when no experimental data is available), based on the thresholds observed in Figure 2 of the main text.

Table II. **Stability data for high-entropy carbonitrides.** The carbonitride compositions (AFLOW prototype AB_cF8_225_a_b) [1–3] are sorted by DEED. DEED and $1/\sigma$ (EFA) are in units of $(\mathrm{eV/atom})^{-1}$, Θ in Kelvin, $\langle \Delta H_{\mathrm{hull}} \rangle$ in (meV/atom), and VEC in (e/cell). Experimental results showing single- and multi-phase forming systems are designated with ● and ✖, respectively, while mixed reports are designated with ■. The **valid.** label refers to results obtained in this work. The (c) in the DEED column indicates that cPOCC was used to parameterize the compound.

carbonitrides													
**composition**	**DEED**	Θ	$\langle \Delta H_{\mathrm{hull}} \rangle$	$1/\sigma$	**VEC**	**exp./pr.**	**composition**	**DEED**	Θ	$\langle \Delta H_{\mathrm{hull}} \rangle$	$1/\sigma$	**VEC**	**exp./pr.**
**3-metal systems**							(HfNbTaTiW)CN	11(c)	1,070	279	33	9.30	✖
(HfTiZr)CN	36	326	91	116	8.50	● [20]	(MoTaTiVZr)CN	11(c)	1,072	287	34	9.30	✖
(HfNbZr)CN	17	680	137	40	8.83	●	(HfMoTiVZr)CN	11(c)	1,073	265	31	9.10	✖
(NbTaTi)CN	17	686	186	53	9.17	●	(HfNbTiWZr)CN	11(c)	1,084	252	29	9.10	✖
(HfTaTi)CN	15	759	146	34	8.83	●	(CrHfMoNbTa)CN	11(c)	1,087	343	39	9.70	✖
(TaTiZr)CN	14	818	161	32	8.83	●	(HfMoNbVZr)CN	11(c)	1,090	290	33	9.30	✖
**4-metal systems**							(CrHfMoNbV)CN	11(c)	1,090	321	36	9.70	✖
(HfNbTiZr)CN	16(c)	736	146	36	8.75	● [20]	(NbTiVWZr)CN	11(c)	1,102	273	30	9.30	✖
(NbTaTiZr)CN	13(c)	910	183	30	9.00	●	(NbTaTiWZr)CN	11(c)	1,104	289	32	9.30	✖
**5-metal systems**							(CrMoTaVW)CN	10(c)	1,109	435	48	10.10	✖ [21]
(CrNbTaTiV)CN	16(c)	719	260	68	9.50	● [21]	(HfTaTiVW)CN	10(c)	1,147	296	30	9.30	✖
(HfNbTaTiZr)CN	15(c)	768	182	41	8.90	● [8, 20–22]	(HfMoTaVZr)CN	10(c)	1,150	313	32	9.30	✖
(HfNbTiVZr)CN	15(c)	774	199	45	8.90	● **valid.**	(HfNbTaVW)CN	10(c)	1,151	346	35	9.50	✖
(HfNbTaTiV)CN	15(c)	793	206	44	9.10	● **valid.**	(HfTaTiWZr)CN	10(c)	1,157	279	28	9.10	✖ **valid.**
(CrHfNbTiZr)CN	14(c)	822	233	46	9.10	● [21]	(HfNbTaWZr)CN	10(c)	1,168	319	31	9.30	✖
(MoNbTaTiV)CN	14(c)	831	273	53	9.50	●	(HfMoNbTiW)CN	10(c)	1,177	303	29	9.50	✖
(NbTaTiVZr)CN	14(c)	835	220	42	9.10	● **valid.**	(MoNbTiWZr)CN	10(c)	1,181	315	30	9.50	✖ **valid.**
(CrHfNbTaTi)CN	14(c)	847	239	45	9.30	✖ [21]	(NbTaVWZr)CN	10(c)	1,187	358	34	9.50	✖
(HfTaTiVZr)CN	14(c)	854	220	41	8.90	● **valid.**	(TaTiVWZr)CN	10(c)	1,189	310	30	9.30	✖
(CrHfTaTiZr)CN	13(c)	892	251	42	9.10	● [21]	(HfTiVWZr)CN	10(c)	1,190	284	27	9.10	✖ **valid.**
(HfNbTaVZr)CN	13(c)	918	251	40	9.10	●	(HfMoNbTaW)CN	10(c)	1,207	393	36	9.70	✖ **valid.**
(HfMoNbTiV)CN	12(c)	934	231	36	9.30	●	(HfNbVWZr)CN	9(c)	1,225	321	29	9.30	✖
(HfMoNbTaTi)CN	12(c)	947	249	37	9.30	●	(HfMoTiVW)CN	9(c)	1,226	316	28	9.50	✖
(HfMoNbTiZr)CN	12(c)	952	226	34	9.10	●	(HfMoNbVW)CN	9(c)	1,227	360	32	9.70	✖
(NbTaTiVW)CN	12(c)	967	304	44	9.50	●	(MoNbTaWZr)CN	9(c)	1,230	401	36	9.70	✖
(MoNbTiVW)CN	12(c)	971	317	45	9.70	●	(HfMoTaTiW)CN	9(c)	1,237	348	31	9.50	✖
(MoNbTaTiZr)CN	12(c)	980	260	36	9.30	● **valid.**	(MoNbVWZr)CN	9(c)	1,255	374	32	9.70	✖
(MoNbTiVZr)CN	12(c)	981	248	35	9.30	✖	(MoTiVWZr)CN	9(c)	1,269	333	28	9.50	✖
(HfMoTaTiZr)CN	11(c)	1,023	250	32	9.10	✖	(MoTaTiWZr)CN	9(c)	1,270	359	30	9.50	✖
(HfMoNbTaV)CN	11(c)	1,027	311	40	9.50	✖	(HfMoNbWZr)CN	9(c)	1,284	355	29	9.50	✖
(MoNbTaTiW)CN	11(c)	1,029	359	46	9.70	✖	(HfTaVWZr)CN	9(c)	1,290	348	28	9.30	✖
(HfMoTaTiV)CN	11(c)	1,030	271	34	9.30	✖	(HfMoTaVW)CN	9(c)	1,305	405	32	9.70	✖
(HfMoNbTaZr)CN	11(c)	1,033	279	35	9.30	✖	(HfMoTiWZr)CN	9(c)	1,310	321	25	9.30	✖
(HfNbTiVW)CN	11(c)	1,057	258	31	9.30	✖	(MoTaVWZr)CN	9(c)	1,336	418	32	9.70	✖
(MoTaTiVW)CN	11(c)	1,057	361	44	9.70	✖	(HfMoTaWZr)CN	9(c)	1,338	384	29	9.50	✖
(MoNbTaVZr)CN	11(c)	1,061	323	39	9.50	✖	(HfMoVWZr)CN	8(c)	1,415	384	26	9.50	✖
(MoNbTaVW)CN	11(c)	1,061	414	50	9.90	✖							

Supplementary Table 3. Synthesizability data and predictions for high-entropy borides

Table III presents synthesizability data for all borides calculated in the aflow.org repository. The dataset includes DEED, compensation temperature Θ, ensemble enthalpy cost $\langle \Delta H_{\mathrm{hull}} \rangle$, ensemble entropy gain $1/\sigma$ (EFA) and VEC. In addition, the single- and multi-phase classifications (●/✖) are reported from the literature or predicted (when no experimental data is available), based on the thresholds observed in Figure 2 of the main text.

Table III. **Stability data for high-entropy borides.** The boride compositions (AFLOW prototype AB2_hP3_191_a_d) [1–3] are sorted by DEED. DEED and $1/\sigma$ (EFA) are in units of $(\mathrm{eV/atom})^{-1}$, Θ in Kelvin, $\langle \Delta H_{\mathrm{hull}} \rangle$ in (meV/atom), and VEC in (e/cell). Experimental results showing single- and multi-phase forming systems are designated with ● and ✖, respectively, while mixed reports are designated with ■. The **valid.** label refers to results obtained in this work.

borides													
**composition**	**DEED**	**Θ**	$\langle \Delta H_{\mathrm{hull}} \rangle$	$1/\sigma$	**VEC**	**exp./pr.**	**composition**	**DEED**	**Θ**	$\langle \Delta H_{\mathrm{hull}} \rangle$	$1/\sigma$	**VEC**	**exp./pr.**
**4-metal systems**							$(HfMoNbVZr)B_2$	46	252	63	134	10.80	●
$(HfNbTaTi)B_2$	175	66	8	251	10.50	● [23]	$(CrNbTaVW)B_2$	45	259	81	163	11.40	●
**5-metal systems**							$(MoNbTaTiW)B_2$	44	264	86	166	11.20	●
$(HfNbTaTiZr)B_2$	126	92	15	239	10.40	● [11, 24–29]	$(HfTaTiVW)B_2$	44	265	72	138	10.80	●
$(HfMoNbTaZr)B_2$	89	131	38	302	10.80	● **valid.**	$(MoNbTiVW)B_2$	44	267	86	163	11.20	●
$(HfNbTaTiV)B_2$	89	131	25	200	10.60	● **valid.**	$(CrHfMoTaTi)B_2$	43	268	70	131	11.00	●
$(MoNbTaTiV)B_2$	77	151	43	253	11.00	●	$(CrHfNbTaZr)B_2$	43	269	45	84	10.80	●
$(HfMoNbTaTi)B_2$	75	155	40	224	10.80	● [26, 29–31]	$(CrNbTaTiZr)B_2$	43	269	50	92	10.80	●
$(MoNbTaTiZr)B_2$	71	164	44	220	10.80	● [29]	$(CrHfNbTaV)B_2$	43	269	49	90	11.00	●
$(NbTaTiVZr)B_2$	68	170	31	146	10.60	●	$(NbTiVWZr)B_2$	43	269	75	140	10.80	●
$(HfNbTaVZr)B_2$	68	171	29	135	10.60	●	$(HfMoTiVZr)B_2$	43	270	66	122	10.60	●
$(HfMoNbTiZr)B_2$	67	173	46	209	10.60	■ [26, 29, 31, 32]	$(CrHfMoNbTi)B_2$	43	271	68	125	11.00	●
$(HfMoTaTiZr)B_2$	64	180	48	200	10.60	● [25, 28, 29, 33]	$(HfMoNbVW)B_2$	42	273	101	182	11.20	●
$(HfMoNbTaV)B_2$	62	186	51	197	11.00	●	$(HfNbVWZr)B_2$	42	275	81	144	10.80	●
$(HfNbTaWZr)B_2$	62	188	60	228	10.80	■ [34]	$(CrHfMoNbTa)B_2$	42	276	65	115	11.20	●
$(HfNbTaYZr)B_2$	58	200	30	100	10.20	●	$(CrHfTaTiV)B_2$	42	276	58	102	10.80	●
$(HfMoNbTiV)B_2$	58	202	53	176	10.80	●	$(HfTaVWZr)B_2$	42	280	79	137	10.80	●
$(HfMoTaTiV)B_2$	56	208	54	169	10.80	●	$(HfMoNbWZr)B_2$	41	280	91	155	11.00	●
$(HfTaTiVZr)B_2$	55	210	42	129	10.40	● [28]	$(HfTiVWZr)B_2$	41	282	80	135	10.60	●
$(MoNbTaVZr)B_2$	53	218	55	156	11.00	●	$(MoTaTiVW)B_2$	41	286	90	149	11.20	●
$(MoNbTaVW)B_2$	53	219	94	266	11.40	●	$(HfMoTaVW)B_2$	41	286	101	166	11.20	●
$(CrMoNbTaTi)B_2$	53	220	57	161	11.20	●	$(TaTiVWZr)B_2$	41	287	78	128	10.80	●
$(NbTaTiVW)B_2$	53	221	62	172	11.00	●	$(HfMoTaWZr)B_2$	40	287	91	148	11.00	●
$(HfNbTiVZr)B_2$	52	223	43	115	10.40	●	$(CrHfNbTiV)B_2$	40	291	58	93	10.80	●
$(HfNbTaVW)B_2$	52	224	72	193	11.00	●	$(HfMoNbTiW)B_2$	40	293	86	135	11.00	●
$(HfNbTaTiW)B_2$	51	228	60	157	10.80	●	$(CrMoTiVW)B_2$	40	293	93	147	11.40	● **valid.**
$(HfNbTiWZr)B_2$	51	228	63	163	10.60	●	$(MoNbTiWZr)B_2$	40	293	90	142	11.00	●
$(CrHfNbTaTi)B_2$	51	230	44	111	10.80	●	$(MoNbVWZr)B_2$	39	295	105	164	11.20	●
$(NbTaTiWZr)B_2$	50	232	64	159	10.80	●	$(CrHfNbTiW)B_2$	39	296	84	129	11.00	●
$(CrMoNbTaV)B_2$	49	238	62	147	11.40	●	$(CrHfMoTiV)B_2$	39	298	75	114	11.00	●
$(MoNbTiVZr)B_2$	48	241	60	139	10.80	●	$(CrMoNbTiW)B_2$	38	302	99	147	11.40	●
$(MoTaTiVZr)B_2$	48	242	61	140	10.80	●	$(CrHfNbTaW)B_2$	38	302	85	125	11.20	●
$(HfMoTaVZr)B_2$	48	242	61	139	10.80	●	$(MoTaVWZr)B_2$	38	303	105	154	11.20	●
$(HfNbTiVW)B_2$	48	244	69	157	10.80	●	$(HfMoTaTiW)B_2$	38	309	90	127	11.00	●
$(MoNbTaWZr)B_2$	47	245	91	205	11.20	●	$(MoTaTiWZr)B_2$	38	309	94	133	11.00	●
$(HfTaTiWZr)B_2$	47	249	66	145	10.60	● [28]	$(CrMoNbTiZr)B_2$	37	312	75	104	11.00	●
$(CrMoNbTaW)B_2$	46	251	105	226	11.60	●	$(CrMoTaTiW)B_2$	37	314	103	140	11.40	■ [34]
$(HfMoNbTaW)B_2$	46	252	89	190	11.20	●	$(CrHfMoTaV)B_2$	36	320	77	101	11.20	●
$(NbTaVWZr)B_2$	46	252	75	160	11.00	●	$(HfMoTiVW)B_2$	36	321	92	121	11.00	●

borides													
**composition**	**DEED**	Θ	$\langle \Delta H_{\mathrm{hull}} \rangle$	$1/\sigma$	**VEC**	**exp./pr.**	**composition**	**DEED**	Θ	$\langle \Delta H_{\mathrm{hull}} \rangle$	$1/\sigma$	**VEC**	**exp./pr.**
(CrHfTaTiZr)B_2	36	322	63	81	10.60	● [25, 28, 29, 31]	(MnMoNbTaZr)B_2	26	439	84	58	11.40	✖
(HfMoTiWZr)B_2	35	328	89	111	10.80	■ [25, 28, 29, 34]	(HfMnNbTaV)B_2	26	440	69	48	11.20	✖
(CrHfMoNbV)B_2	35	328	79	98	11.20	●	(MnMoTaVW)B_2	26	442	119	82	11.80	✖
(CrNbTiWZr)B_2	35	329	90	112	11.00	●	(HfMnNbTiV)B_2	26	452	75	50	11.00	✖
(CrHfNbTiZr)B_2	35	333	63	77	10.60	● **valid.**	(HfMnTaTiV)B_2	26	455	77	50	11.00	✖
(CrHfMoTaZr)B_2	35	333	77	93	11.00	✖	(HfMnTiVW)B_2	25	460	105	67	11.20	✖
(CrHfMoTiZr)B_2	35	333	83	101	10.80	✖	(HfMnMoTaW)B_2	25	465	125	78	11.60	✖
(CrHfNbVW)B_2	34	337	95	113	11.20	✖	(HfMoTaVY)B_2	25	466	96	59	10.60	✖
(MnNbTaTiV)B_2	34	339	54	64	11.20	✖	(HfMnMoNbW)B_2	25	466	122	76	11.60	✖
(MoTiVWZr)B_2	34	340	99	116	11.00	✖	(MnMoNbTiZr)B_2	25	466	92	57	11.20	✖
(CrHfMoNbW)B_2	34	341	114	132	11.40	✖	(HfMoWYZr)B_2	25	466	114	71	10.60	✖
(HfMoVWZr)B_2	34	344	111	126	11.00	✖	(MnNbTaWZr)B_2	25	467	105	65	11.40	✖
(CrHfMoTaW)B_2	34	346	113	127	11.40	✖ **valid.**	(MnMoTaTiZr)B_2	25	468	94	58	11.20	✖
(CrHfMoNbZr)B_2	33	347	80	89	11.00	✖	(MnNbTiWZr)B_2	25	472	105	64	11.20	✖
(CrNbTiVZr)B_2	33	352	68	73	10.80	✖	(MnTaTiWZr)B_2	25	472	107	65	11.20	✖
(MnMoNbTaTi)B_2	32	359	71	74	11.40	✖	(HfMnNbTaZr)B_2	24	480	72	42	11.00	✖
(CrHfNbWZr)B_2	32	363	96	98	11.00	✖	(HfMnMoTiV)B_2	24	481	93	54	11.20	✖
(CrHfTaWZr)B_2	32	363	94	96	11.00	✖	(HfMnTaVW)B_2	24	483	111	64	11.40	✖
(NbTaWYZr)B_2	32	364	82	83	10.60	✖	(HfMnMoTaV)B_2	24	489	94	53	11.40	✖
(CrMoTaVZr)B_2	32	364	84	85	11.20	✖	(MnNbTaVZr)B_2	24	490	78	44	11.20	✖
(MnMoNbTiV)B_2	31	369	74	73	11.40	✖	(HfMnNbVW)B_2	24	492	114	63	11.40	✖
(CrHfMoTiW)B_2	31	370	107	105	11.20	✖	(HfMnMoTiW)B_2	24	494	118	65	11.40	✖
(MoNbTaTiY)B_2	31	372	77	75	10.60	✖	(HfMnMoNbV)B_2	23	499	96	52	11.40	✖
(NbTaTiVY)B_2	31	374	56	54	10.40	✖	(HfMnMoTaZr)B_2	23	501	94	50	11.20	✖
(MnMoTaTiV)B_2	31	376	76	73	11.40	✖	(MnMoTaWZr)B_2	23	503	129	69	11.60	✖
(MnNbTaTiW)B_2	31	378	86	81	11.40	✖	(MnTaTiVZr)B_2	23	504	85	45	11.00	✖
(CrHfTaVZr)B_2	31	378	69	65	10.80	✖	(MnMoNbWZr)B_2	23	507	131	68	11.60	✖
(MnMoTiVW)B_2	31	379	106	99	11.60	✖	(HfMnMoNbZr)B_2	23	512	98	50	11.20	✖
(HfMnNbTaTi)B_2	31	379	60	56	11.00	✖	(MnNbTiVZr)B_2	23	514	86	44	11.00	✖
(MnNbTiVW)B_2	30	381	89	82	11.40	✖	(MnTiVWZr)B_2	23	514	114	58	11.20	✖
(CrHfMoVW)B_2	30	382	123	114	11.40	✖	(CrHfNbTaY)B_2	22	517	100	50	10.60	✖
(CrTaVWZr)B_2	30	382	100	93	11.20	✖	(HfMnMoVW)B_2	22	518	136	68	11.60	✖
(HfTiWYZr)B_2	30	382	98	90	10.20	✖	(HfMnTiWZr)B_2	22	521	114	57	11.00	✖
(MnMoNbTaV)B_2	30	382	76	70	11.60	✖	(MnTaVWZr)B_2	22	522	117	58	11.40	✖
(MnTaTiVW)B_2	30	388	90	81	11.40	✖	(HfMnTaVZr)B_2	22	525	86	42	11.00	✖
(MoTaTiYZr)B_2	30	391	85	74	10.40	✖	(HfMnMoTiZr)B_2	22	526	104	50	11.00	✖
(CrHfNbVZr)B_2	29	395	74	63	10.80	✖	(MnMoTaVZr)B_2	22	529	102	49	11.40	✖
(MoNbTaWY)B_2	29	405	117	96	11.00	✖	(MnMoTiWZr)B_2	22	531	129	62	11.40	✖
(HfMnMoNbTa)B_2	29	406	78	63	11.40	✖	(MnMoTiVZr)B_2	22	532	103	49	11.20	✖
(MnNbTaVW)B_2	28	409	95	76	11.60	✖	(HfMnTaWZr)B_2	22	535	116	55	11.20	✖
(HfMnMoNbTi)B_2	28	409	80	64	11.20	✖	(HfMnNbWZr)B_2	21	542	117	53	11.20	✖
(CrHfMoWZr)B_2	28	414	126	99	11.20	✖	(MnNbVWZr)B_2	21	545	121	55	11.40	✖
(MnMoNbTiW)B_2	28	416	108	84	11.60	✖	(CrHfTaYZr)B_2	21	546	111	50	10.40	✖
(HfMnMoTaTi)B_2	28	420	83	63	11.20	✖	(MnMoNbVZr)B_2	21	549	105	47	11.40	✖
(HfMnNbTiW)B_2	28	421	94	71	11.20	✖	(CrMoNbTaY)B_2	21	557	121	53	11.00	✖
(MnNbTaTiZr)B_2	27	422	68	52	11.00	✖	(TiVWYZr)B_2	21	557	127	55	10.40	✖
(MnMoNbTaW)B_2	27	425	115	86	11.80	✖	(MnMoVWZr)B_2	21	561	143	61	11.60	✖
(MnMoTaTiW)B_2	27	427	113	84	11.60	✖	(HfMnNbVZr)B_2	21	562	92	39	11.00	✖
(CrHfVWZr)B_2	27	430	113	83	11.00	✖	(CrHfNbYZr)B_2	21	562	117	50	10.40	✖
(HfMnNbTaW)B_2	27	433	101	73	11.40	✖	(HfMnMoWZr)B_2	20	569	138	57	11.40	✖
(CrHfTiVZr)B_2	27	433	84	60	10.60	✖	(HfMnTaTiZr)B_2	20	571	91	38	10.80	✖
(MnMoNbVW)B_2	27	434	119	85	11.80	✖	(CrHfMoTaY)B_2	20	571	128	53	10.80	✖
(CrHfMoVZr)B_2	27	435	100	71	11.00	✖	(HfMnNbTiZr)B_2	20	580	91	37	10.80	✖
(HfMnTaTiW)B_2	27	436	98	70	11.20	✖	(CrHfMoNbY)B_2	20	587	133	52	10.80	✖

**borides**													
**composition**	**DEED**	Θ	$\langle \Delta H_{\mathrm{hull}} \rangle$	$1/\sigma$	**VEC**	**exp./pr.**	**composition**	**DEED**	Θ	$\langle \Delta H_{\mathrm{hull}} \rangle$	$1/\sigma$	**VEC**	**exp./pr.**
$(CrHfMoYZr)B_2$	19	597	146	55	10.60	✖	$(HfIrMoNbV)B_2$	8	1,368	1,322	95	11.60	✖
$(CrHfTaWY)B_2$	19	597	143	54	10.80	✖	$(IrMoNbVW)B_2$	8	1,370	1,367	98	12.00	✖
$(CrHfWYZr)B_2$	19	600	157	59	10.60	✖	$(HfIrNbWZr)B_2$	8	1,372	1,329	95	11.40	✖
$(CrHfNbWY)B_2$	19	602	147	54	10.80	✖	$(HfIrMoNbW)B_2$	8	1,388	1,357	95	11.80	✖
$(HfMnVWZr)B_2$	19	603	134	50	11.20	✖	$(HfIrTiVW)B_2$	8	1,390	1,344	94	11.40	✖
$(CrHfNbTiY)B_2$	19	616	129	46	10.40	✖	$(HfIrTiWZr)B_2$	8	1,393	1,335	93	11.20	✖
$(HfMnMoVZr)B_2$	19	618	121	43	11.20	✖	$(IrNbTaVW)B_2$	8	1,397	1,339	92	11.80	✖
$(CrTaTiWY)B_2$	19	623	150	52	10.80	✖	$(HfIrMoTaV)B_2$	8	1,398	1,320	91	11.60	✖
$(CrHfMoTiY)B_2$	19	627	149	51	10.60	✖	$(IrNbTiWZr)B_2$	8	1,404	1,334	91	11.40	✖
$(CrTaTiYZr)B_2$	18	628	131	45	10.40	✖	$(HfIrTaTiZr)B_2$	8	1,414	1,295	87	11.00	✖
$(CrTiWYZr)B_2$	18	647	163	52	10.60	✖	$(HfIrTaWZr)B_2$	8	1,417	1,327	89	11.40	✖
$(CrHfMoWY)B_2$	18	660	175	54	11.00	✖	$(HfIrMoTaW)B_2$	8	1,419	1,358	91	11.80	✖
$(HfMnTiVZr)B_2$	17	674	109	32	10.80	✖ **valid.**	$(IrMoTaVW)B_2$	8	1,421	1,367	91	12.00	✖
$(CrHfTiYZr)B_2$	17	681	148	43	10.20	✖ **valid.**	$(HfIrNbTaV)B_2$	8	1,430	1,294	85	11.40	✖
$(CrTaVWY)B_2$	17	682	156	45	11.00	✖	$(HfIrNbVW)B_2$	8	1,433	1,346	88	11.60	✖
$(CrNbVWY)B_2$	17	693	160	45	11.00	✖	$(IrMoNbWZr)B_2$	8	1,443	1,359	88	11.80	✖
$(CrTaTiVY)B_2$	17	695	137	38	10.60	✖	$(IrTaTiWZr)B_2$	8	1,445	1,337	86	11.40	✖
$(CrHfNbVY)B_2$	17	702	139	38	10.60	✖ **valid.**	$(HfIrMoVW)B_2$	8	1,450	1,374	88	11.80	✖
$(CrNbTiVY)B_2$	16	717	140	37	10.60	✖	$(IrMoTaWZr)B_2$	8	1,460	1,359	86	11.80	✖
$(CrHfVWY)B_2$	16	728	174	44	10.80	✖	$(IrMoNbVZr)B_2$	8	1,472	1,326	82	11.60	✖
$(CrHfMoVY)B_2$	16	731	163	41	10.80	✖	$(HfIrTaVW)B_2$	8	1,474	1,345	83	11.60	✖
$(CrVWYZr)B_2$	15	754	178	42	10.80	✖	$(HfIrMoTiW)B_2$	8	1,488	1,354	82	11.60	✖
$(CrMoVYZr)B_2$	15	758	169	39	10.80	✖	$(IrMoTiVZr)B_2$	8	1,489	1,327	81	11.40	✖
$(CrTiVYZr)B_2$	14	803	164	34	10.40	✖	$(HfIrMoVZr)B_2$	8	1,490	1,332	81	11.40	✖
$(IrMoNbTaTi)B_2$	11	1,099	1,304	145	11.60	✖	$(HfIrNbTiV)B_2$	8	1,490	1,304	79	11.20	✖
$(HfIrMoNbTa)B_2$	10	1,165	1,301	129	11.60	✖	$(IrMoTaVZr)B_2$	8	1,495	1,324	80	11.60	✖
$(HfIrMoNbTi)B_2$	10	1,173	1,308	128	11.40	✖	$(HfIrTaTiV)B_2$	8	1,517	1,305	76	11.20	✖
$(HfIrMoTaTi)B_2$	10	1,208	1,311	121	11.40	✖	$(IrNbVWZr)B_2$	8	1,539	1,349	77	11.60	✖
$(IrNbTaTiW)B_2$	10	1,216	1,329	121	11.60	✖	$(IrNbTaVZr)B_2$	8	1,539	1,297	74	11.40	✖
$(HfIrMoTiZr)B_2$	9	1,236	1,312	116	11.20	✖	$(IrTiVWZr)B_2$	8	1,542	1,349	76	11.40	✖
$(HfIrMoNbZr)B_2$	9	1,246	1,305	113	11.40	✖	$(IrMoVWZr)B_2$	8	1,545	1,378	78	11.80	✖
$(IrMoNbTiV)B_2$	9	1,247	1,314	114	11.60	✖	$(IrMoTiWZr)B_2$	7	1,559	1,357	75	11.60	✖
$(HfIrNbTaTi)B_2$	9	1,247	1,284	111	11.20	✖	$(IrTaVWZr)B_2$	7	1,578	1,348	73	11.60	✖
$(IrMoNbTaW)B_2$	9	1,254	1,358	116	12.00	✖	$(HfIrNbVZr)B_2$	7	1,591	1,306	69	11.20	✖
$(HfIrMoTaZr)B_2$	9	1,257	1,303	111	11.40	✖	$(HfIrVWZr)B_2$	7	1,595	1,354	72	11.40	✖
$(IrMoNbTaZr)B_2$	9	1,258	1,302	111	11.60	✖	$(IrNbTiVZr)B_2$	7	1,599	1,309	69	11.20	✖
$(IrMoNbTiW)B_2$	9	1,271	1,354	113	11.80	✖	$(HfIrMoWZr)B_2$	7	1,618	1,358	70	11.60	✖
$(HfIrNbTaW)B_2$	9	1,283	1,327	109	11.60	✖	$(HfIrTaVZr)B_2$	7	1,622	1,304	67	11.20	✖
$(HfIrNbTiW)B_2$	9	1,287	1,331	108	11.40	✖	$(IrTaTiVZr)B_2$	7	1,636	1,310	66	11.20	✖
$(IrMoNbTiZr)B_2$	9	1,288	1,311	106	11.40	✖	$(HfIrTiVZr)B_2$	7	1,646	1,316	65	11.00	✖
$(IrMoTaTiV)B_2$	9	1,292	1,317	106	11.60	✖	$(IrMnMoNbTa)B_2$	7	1,665	1,349	66	12.20	✖
$(HfIrNbTaZr)B_2$	9	1,296	1,275	102	11.20	✖	$(IrMnMoTiW)B_2$	7	1,666	1,386	67	12.20	✖
$(IrNbTiVW)B_2$	9	1,301	1,337	106	11.60	✖	$(IrMnNbTaW)B_2$	7	1,690	1,366	64	12.20	✖
$(IrMoTaTiW)B_2$	9	1,314	1,358	106	11.80	✖	$(IrMnTiVW)B_2$	7	1,698	1,369	64	12.00	✖
$(IrMoTaTiZr)B_2$	9	1,323	1,313	101	11.40	✖	$(IrMnMoTiV)B_2$	7	1,699	1,355	63	12.00	✖
$(IrMoNbTaV)B_2$	9	1,324	1,314	101	11.80	✖	$(IrMnMoNbW)B_2$	7	1,707	1,393	64	12.40	✖
$(IrNbTaTiV)B_2$	9	1,327	1,293	99	11.40	✖	$(IrMnMoNbTi)B_2$	7	1,708	1,352	62	12.00	✖
$(HfIrTaTiW)B_2$	9	1,331	1,335	101	11.40	✖	$(IrMnNbTiW)B_2$	7	1,717	1,366	62	12.00	✖
$(HfIrMoTiV)B_2$	9	1,341	1,322	99	11.40	✖	$(IrMnMoTaTi)B_2$	7	1,739	1,355	60	12.00	✖
$(IrNbTaTiZr)B_2$	9	1,354	1,287	95	11.20	✖	$(IrMnMoNbV)B_2$	7	1,769	1,360	59	12.20	✖
$(IrTaTiVW)B_2$	9	1,354	1,340	98	11.60	✖	$(HfIrMnMoTi)B_2$	7	1,775	1,361	58	11.80	✖
$(IrMoTiVW)B_2$	9	1,362	1,358	99	11.80	✖	$(IrMnMoTaV)B_2$	6	1,790	1,358	57	12.20	✖
$(IrNbTaWZr)B_2$	9	1,363	1,328	96	11.60	✖	$(HfIrMnMoTa)B_2$	6	1,795	1,359	57	12.00	✖
$(HfIrNbTiZr)B_2$	8	1,367	1,293	93	11.00	✖	$(HfIrMnTiW)B_2$	6	1,796	1,376	57	11.80	✖

**borides**													
**composition**	**DEED**	Θ	$\langle\Delta H_{\text{hull}}\rangle$	$1/\sigma$	**VEC**	**exp./pr.**	**composition**	**DEED**	Θ	$\langle\Delta H_{\text{hull}}\rangle$	$1/\sigma$	**VEC**	**exp./pr.**
$(HfIrMnMoW)B_2$	6	1,805	1,401	58	12.20	✖	$(HfIrMnNbTa)B_2$	6	1,925	1,332	48	11.80	✖
$(IrMnMoTaW)B_2$	6	1,805	1,394	58	12.40	✖	$(IrMnTaVW)B_2$	6	1,929	1,373	50	12.20	✖
$(HfIrMnMoNb)B_2$	6	1,820	1,359	55	12.00	✖	$(HfIrMnTaTi)B_2$	6	1,943	1,347	48	11.60	✖
$(HfIrMnTaW)B_2$	6	1,827	1,374	55	12.00	✖	$(HfIrMnTiV)B_2$	6	1,953	1,355	48	11.60	✖
$(IrMnNbTaTi)B_2$	6	1,843	1,333	53	11.80	✖	$(IrMnVWZr)B_2$	6	1,957	1,395	49	12.00	✖
$(HfIrMnMoV)B_2$	6	1,845	1,374	54	12.00	✖	$(HfIrMnNbTi)B_2$	6	1,981	1,345	46	11.60	✖
$(IrMnMoTiZr)B_2$	6	1,845	1,367	54	11.80	✖	$(IrMnNbTaZr)B_2$	6	2,006	1,335	45	11.80	✖
$(HfIrMnNbW)B_2$	6	1,858	1,374	54	12.00	✖	$(HfIrMnMoZr)B_2$	6	2,013	1,373	46	11.80	✖
$(IrMnNbVW)B_2$	6	1,867	1,374	53	12.20	✖	$(HfIrMnNbV)B_2$	6	2,045	1,352	44	11.80	✖
$(IrMnMoNbZr)B_2$	6	1,873	1,363	52	12.00	✖	$(HfIrMnTaV)B_2$	6	2,050	1,349	43	11.80	✖
$(HfIrMnVW)B_2$	6	1,882	1,391	53	12.00	✖	$(IrMnMoWZr)B_2$	6	2,052	1,408	45	12.20	✖
$(IrMnMoVW)B_2$	6	1,890	1,399	53	12.40	✖	$(IrMnNbTiZr)B_2$	6	2,053	1,349	43	11.60	✖
$(IrMnNbTiV)B_2$	6	1,891	1,340	50	11.80	✖	$(IrMnTaTiZr)B_2$	6	2,073	1,351	42	11.60	✖
$(IrMnNbTaV)B_2$	6	1,893	1,336	50	12.00	✖	$(HfIrMnWZr)B_2$	6	2,083	1,385	43	11.80	✖
$(IrMnMoTaZr)B_2$	6	1,894	1,361	51	12.00	✖	$(IrMnTiVZr)B_2$	6	2,091	1,362	42	11.60	✖
$(IrMnTaTiV)B_2$	6	1,896	1,341	50	11.80	✖	$(HfIrMnTaZr)B_2$	5	2,135	1,342	40	11.60	✖
$(IrMnTaTiW)B_2$	6	1,906	1,369	51	12.00	✖	$(HfIrMnNbZr)B_2$	5	2,139	1,345	40	11.60	✖
$(IrMnTiWZr)B_2$	6	1,909	1,382	51	11.80	✖	$(IrMnNbVZr)B_2$	5	2,147	1,357	40	11.80	✖
$(IrMnTaWZr)B_2$	6	1,920	1,377	50	12.00	✖	$(IrMnTaVZr)B_2$	5	2,158	1,354	39	11.80	✖
$(IrMnMoVZr)B_2$	6	1,921	1,381	50	12.00	✖	$(HfIrMnTiZr)B_2$	5	2,189	1,359	38	11.40	✖
$(IrMnNbWZr)B_2$	6	1,924	1,378	50	12.00	✖	$(HfIrMnVZr)B_2$	5	2,262	1,369	36	11.60	✖

Supplementary Discussion 1. Experimental considerations for high-entropy carbonitrides

We have evaluated the synthesis of high-entropy carbonitrides across various soak conditions to determine the extent of the entropy stabilization. For example, in Fig. 1**a-b**, the constituent six individual powders consolidated into two cubic solid solution phases at 2,200 °C/30 min and then stabilized into one cubic single phase at 2,300 °C/60 min. This highlights the influence of thermokinetics on entropy-stabilized phases, as substantial diffusion and chemical driving forces are needed to facilitate the coalescence of these different species into a single disordered crystalline phase. In Fig. 1**c-d**, more homogenous multiphase mixtures were observed at the 2,300 °C/60 min soak relative to 2,200 °C/30 min, reinforcing that these multiphase compositions cannot be further stabilized into even fewer phases at higher soak conditions.

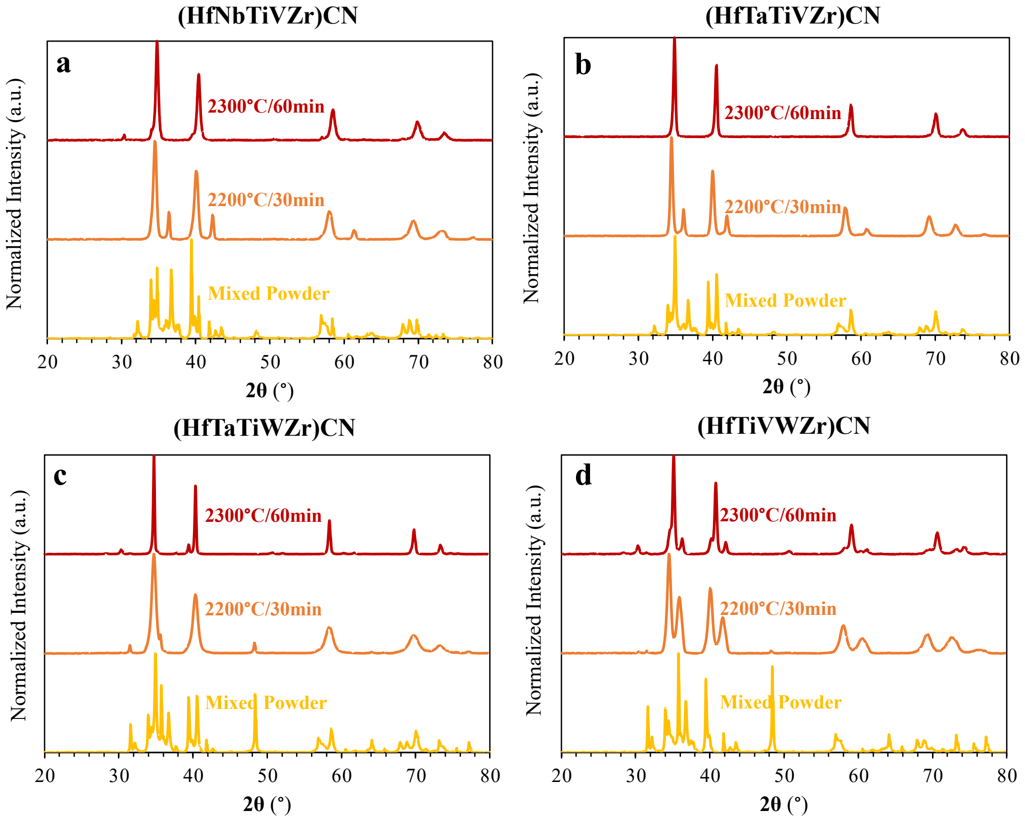

Fig. 1. X-ray diffraction comparison of high-entropy carbonitride phase evolution from mixed powders across soak conditions of (**a**) (HfNbTiVZr)CN, (**b**) (HfTaTiVZr)CN, (**c**) (HfTaTiWZr)CN, and (**d**) (HfTiVWZr)CN.

The entropy stabilization is not only invariant to minor changes in relative cation content, but also in anion content as well. As shown in Fig. 2, two identical high-entropy carbonitrides were synthesized under the same processing conditions, but one with 5 at% C added and one without. It is seen that no significant change in phase can be discerned from this minor anion content variation. At most, a slight change in relative peak intensity ($<$5%) may be attributed to the slight differences in carbon stoichiometry. Therefore, the DEED descriptor-based predictions remain accurate despite minor changes in anion content with respect to ideal equi-molarity.

To further examine the phase stability of high-entropy carbonitrides with respect to anion content, carbothermal reduction (CTR) on blended powders was performed to determine subsequent changes on the resulting high-entropy carbonitride. This CTR was performed under pressureless conditions with a modified FAST die assembly (60 mm cavity), where 25 at% C was added and heat treated at 1,450 °C/3 h. These conditions were chosen to simultaneous maximize both the CTR driving forces ($>$1,200 °C) and available open porosity for reaction before densification ($<$1,600 °C). The resulting reacted powder was pulverized/deagglomerated with a mortar and pestle, sieved, and sintered at 2,300 °C/60 min. As seen in Fig. 3, systematic decreases in oxide peak intensity are observed for the post-CTR high-entropy carbonitride and demonstrate oxide reduction. Minor peak intensity changes were observed as previously seen in Fig. 2 (i.e. carbon stoichiometry effect), but the overall phases remained invariant to this change.

To further evaluate the compositional phase segregation the microstructures were examined with SEM and multi-phase elemental maps obtained with EDS. The previously designated cubic phases A/B and hexagonal WC phases

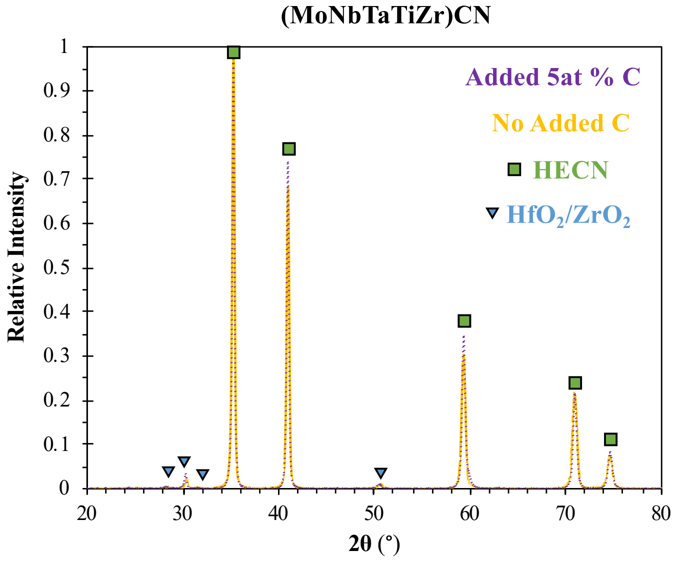

Fig. 2. X-ray diffraction comparison of adding 5 at% trace carbon on a typical synthesized high-entropy carbonitride, (MoNbTa-TiZr)CN, sintered at 2,300 °C/60 min.

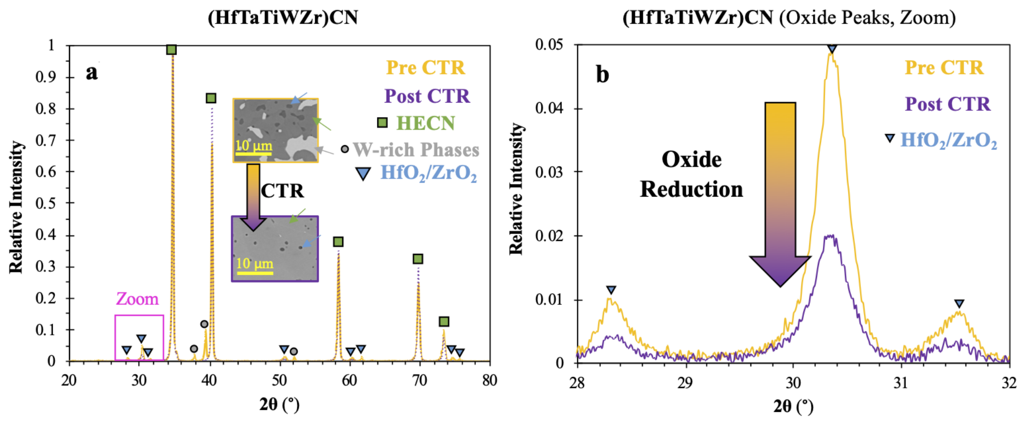

Fig. 3. X-ray diffraction comparison of carbothermal reduction treatment (25 at% C, 1,450 °C/3 h) on a typical high-entropy carbonitride, (HfTaTiWZr)CN sintered at 2,300 °C/60 min across (**a**) full angular range and (**b**) select oxide peaks.

were confirmed accordingly, where phase A constituted the microstructure as the continuous, primary matrix phase and WC/phase B appeared as dispersed secondary phases, both isolated and grain boundary-segregated. Additional oxide inclusions were detected as dispersed phases, likely originating from trace impurities from the source powders (e.g. HfO_2, ZrO_2) and minor abrasive wear from blending with YSZ-based media. Nonetheless, the overall microstructures are composed of coarse carbonitride grains (∼10-50 µm) with minimal pores. In particular with the tungsten-rich compositions, the immiscible multiphase mixture yielded dispersions of partially-dissolved precursor phases throughout the microstructure, indicating the highly unfavorable nature of attempting dissolve these mutually insoluble components into a single phase.

Previous studies of single-phase (HfNbTaTiZr)CN have been reported using hot-pressed sintering [8], spark plasma sintering (SPS) [20, 21], and high-energy ball milling assisted carbothermal reduction nitridation [22]. The preparation of the other carbonitrides has been attempted via SPS [20, 21], yielding single-phase systems, except for (CrHfNbTaTi)CN (chemical segregation) and (CrMoTaVW)CN (decomposed into two phases) [21].

Supplementary Discussion 2. Synthesizability and valence electron concentrations

Figure 4 plots $\langle \Delta H_{\mathrm{hull}} \rangle$ (1st row), $1/\sigma$ (EFA, 2nd row), and DEED (3rd row) vs. VEC for the carbonitrides (1st column), carbides (2nd column), and borides (3rd column). The results suggest that, across all three classes of high-entropy ceramics, VEC correlates with the ensemble enthalpic costs, and not with the entropic gains.

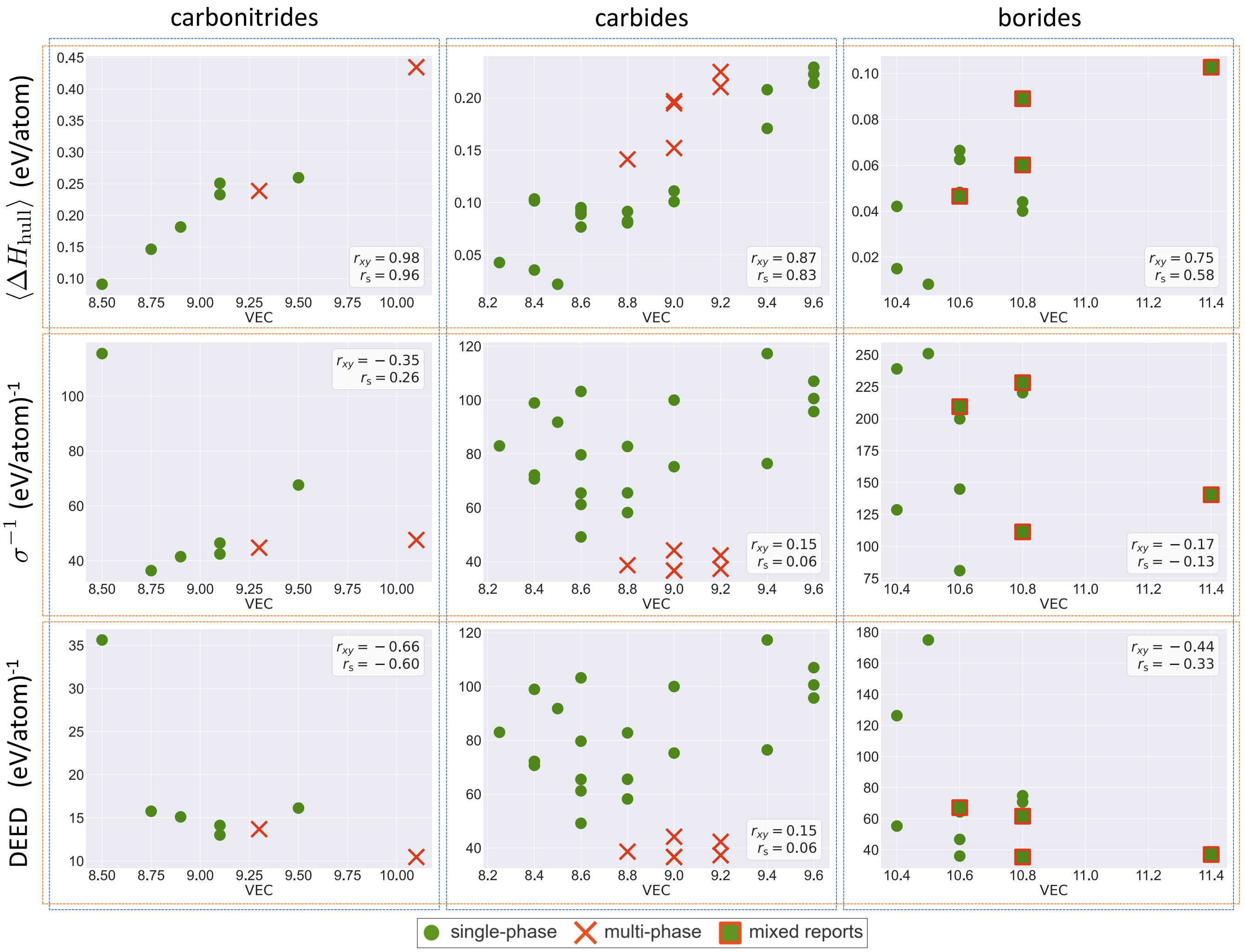

Fig. 4. **Correlating synthesizability descriptors with valence electron concentration.** The ensemble enthalpic cost $\langle \Delta H_{\mathrm{hull}} \rangle$ (1st row), $1/\sigma$ (EFA, 2nd row), and DEED (3rd row) are plotted against VEC for the carbonitrides (1st column), carbides (2nd column), and borides (3rd column). r_{xy} and r_s are the Pearson and the Spearman's rank correlation coefficients, respectively.

Correlations between the ensemble enthalpy cost $\langle \Delta H_{\text{hull}} \rangle$, $1/\sigma$ (EFA), and DEED with the standard deviation of the binary components' VEC are plotted in Fig. 5. The standard deviation correlates with $1/\sigma$ for the carbides, and $\langle \Delta H_{\text{hull}} \rangle$ and DEED for the borides, suggesting a useful pre-screening heuristic.

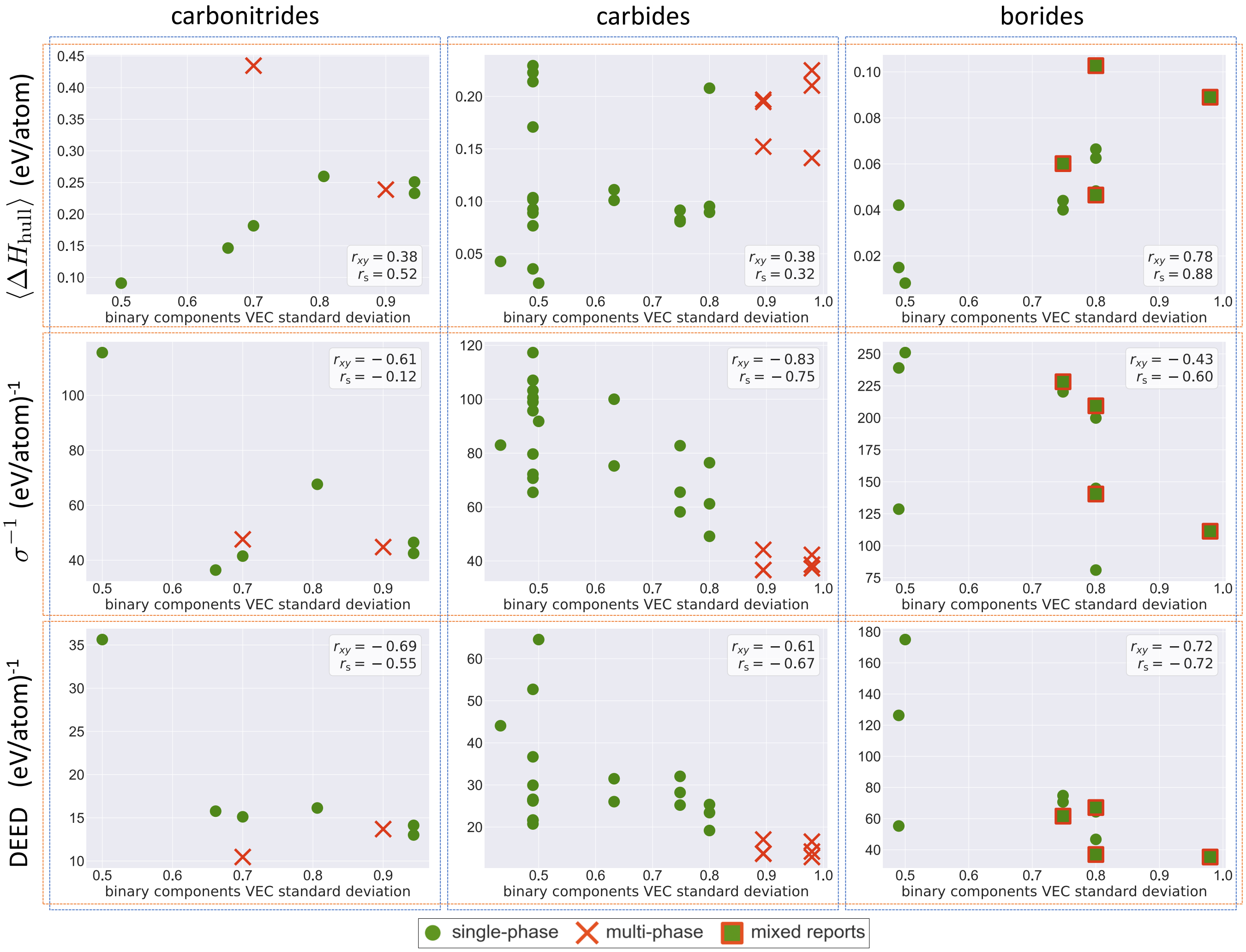

Fig. 5. **Correlating synthesizability descriptors with the standard deviation of the binary components' VEC.** The ensemble enthalpic cost $\langle \Delta H_{\text{hull}} \rangle$ (1st row), $1/\sigma$ (EFA, 2nd row), and DEED (3rd row) are plotted against the standard deviation of the binary components' VEC for the carbonitrides (1st column), carbides (2nd column), and borides (3rd column). r_{xy} is the Pearson correlation coefficient and r_s is Spearman's rank correlation coefficient.

Supplementary Discussion 3. Experimental considerations for high-entropy borides

XRD of the ceramics after SPS is presented in Fig. 3. The Mo-HEB and V-HEB specimens contained single phase with a hexagonal crystal structure, but had slightly different lattice parameters, shown in Table IV. For these compositions, two step SPS at 1,900 °C lead to complete solution. In contrast, the Y-HEB was not a single phase after SPS. This composition contained residual oxide phases and a Y-containing boride phase in addition to a hexagonal boride phase. In addition, the Y-based composition partially melted during SPS. In the Y-B system, various phases can form. Phases that are rich in Y have melting points below 1,900 °C and could have formed in this case.

**Label**	**Composition**	**Lattice parameter** a (Å)	**Lattice parameter** c (Å)	**Vickers hardness** (GPa)
Mo-HEB	$(HfMoNbTaZr)B_2$	3.1044(5)	3.3787(0)	28.5 ± 0.7
V-HEB	$(HfNbTaTiV)B_2$	3.0724(2)	3.2996(4)	20.3 ± 0.2
Y-HEB	$(CrHfTiYZr)B_2$	–	–	23.5 ± 0.6
Y,V-HEB	$(CrHfNbVY)B_2$	–	–	23.8 ± 0.9

Table IV. Tabulated summary of SPS high-entropy boride lattice parameters.

The microstructures of selected HEB ceramics are presented in Fig. 3. The Mo-HEB ceramic was almost full density with a negligible amount of porosity. However, a small fraction of residue carbon was evident as dark inclusions. The average grain size of Mo-HEB was 3.4 ± 1.0 µm. The V-HEB contained some porosity and residual carbon. The grain size was approximately 1.5 ± 0.4 µm. The smaller grain size of V-HEB compared to Mo-HEB was due to the pinning effect of the secondary phases. The microstructure of Y-HEB was more complex and contained multiple phases. No residual carbon or porosity were observed, but the multiple phases indicated that the predicted Y-HEB phase was not formed.

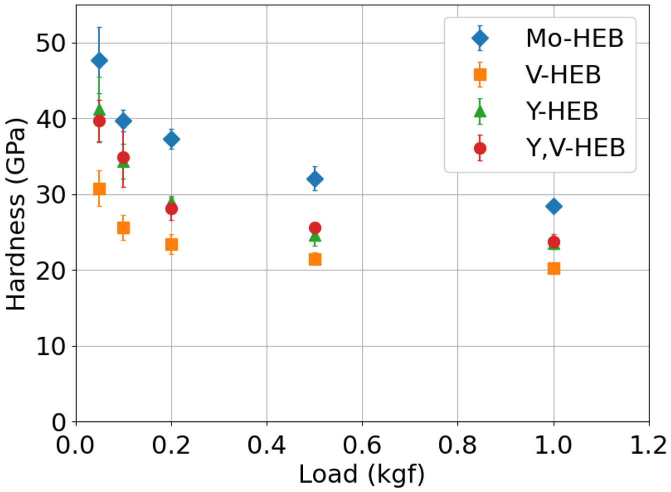

Fig. 6. Vickers hardness dependence on the applied load for SPS ceramics.

The microstructures of the HEB ceramics are presented in Fig. 6. The Mo-HEB ceramic was almost full density with a negligible amount of porosity. However, a small fraction of residue carbon was evident as dark inclusions. The average grain size of Mo-HEB was 3.4 ± 1.0 µm. The V-HEB contained some porosity and residual carbon. The grain size was approximately 1.5 ± 0.4 µm. The smaller grain size of V-HEB compared to Mo-HEB was due to the pinning effect of the secondary phases. The microstructures of Y-HEB and Y,V-HEB were more complex and contained multiple phases. No residual carbon or porosity were observed, but the multiple phases indicated that the predicted Y-HEB and Y,V-HEB phases were not formed. The harnesses values, at a load of 9.81 N, were 28.4 ± 0.7 GPa for Mo-HEB, 20.3 ± 0.2 GPa for V-HEB, 23.5 ± 0.6 GPa for Y-HEB and 23.8 ± 0.9 GPa for Y,V-HEB. The hardness obtained for Mo-HEB is higher than similar compositions reported previously [28, 35]. The higher hardness likely originates from the high phase purity and relative density of the ceramic.

EDS mapping (see Extended Data Fig. 5) for the Mo-HEDB sample shows relatively uniform distribution of all phases. Only slight segregation of the Hf was noticed. V-HEDB composition behaves similar, insignificant segregation of the hafnium was detected. These results are in accordance with XRD analyses, where the single-phase structures were found for these two samples. Contrary, compositions with Y exhibit significant phase separation. It appears

that Hf and Ti segregate within same grains, while Cr and Y segregate separately in the Y-HEDB composition. On the other hand, vanadium addition in the composition denoted as Y,V helps better mixing of the V, Hf, Cr and Nb in one phase, and Y-based boride was distinguished as separate phase.

Previous studies have reported single-phase borides that were prepared via several synthesis methods: e.g., $(HfNbTaTiZr)B_2$ by SPS [11, 29], borothermal reduction [24], and boro/carbothermal reduction with SPS (to promote densification) [25–28]. For different chemistries, the synthesizability has been shown to depend on the experimental route: **i.** $(HfMoNbTiZr)B_2$ forms a single-phase with SPS of binary borides [29], borothermal reduction [31], and boro/carbothermal reduction with SPS [26], whereas SPS of elemental powders yields a multiphase product — an outcome later corrected in the same study by preceding SPS with self-propagating high-temperature synthesis [32]; **ii.** $(HfNbTaWZr)B_2$, $(CrMoTaTiW)B_2$, and $(HfMoTiWZr)B_2$ phase-separate with SPS of the binary metal borides, but form single phases through a multi-stage SPS route [29, 36]; and **iii.** $(HfMoTiWZr)B_2$ additionally phase-separates via boro/carbothermal reduction with SPS [25], and was later shown to form a single-phase with a subsequent two-step sintering (parameters varying) [28].

SUPPLEMENTARY REFERENCES

[1] M. J. Mehl, D. Hicks, C. Toher, O. Levy, R. M. Hanson, G. L. W. Hart, and S. Curtarolo, *The AFLOW Library of Crystallographic Prototypes: Part 1*, Comput. Mater. Sci. **136**, S1–S828 (2017), doi:10.1016/j.commatsci.2017.01.017.

[2] D. Hicks, M. J. Mehl, E. Gossett, C. Toher, O. Levy, R. M. Hanson, G. L. W. Hart, and S. Curtarolo, *The AFLOW Library of Crystallographic Prototypes: Part 2*, Comput. Mater. Sci. **161**, S1–S1011 (2019), doi:10.1016/j.commatsci.2018.10.043.

[3] D. Hicks, M. J. Mehl, M. Esters, C. Oses, O. Levy, G. L. W. Hart, C. Toher, and S. Curtarolo, *The AFLOW Library of Crystallographic Prototypes: Part 3*, Comput. Mater. Sci. **199**, 110450 (2021), doi:10.1016/j.commatsci.2021.110450.

[4] E. Castle, T. Csanádi, S. Grasso, J. Dusza, and M. Reece, *Processing and Properties of High-Entropy Ultra-High Temperature Carbides*, Sci. Rep. **8**, 8609 (2018), doi:10.1038/s41598-018-26827-1.

[5] B. Ye, T. Wen, K. Huang, C.-Z. Wang, and Y. Chu, *First-principles study, fabrication, and characterization of* $(Hf_{0.2}Zr_{0.2}Ta_{0.2}Nb_{0.2}Ti_{0.2})C$ *high-entropy ceramic*, J. Am. Ceram. Soc. **102**, 4344–4352 (2019), doi:10.1111/jace.16295.

[6] F. Li, Y. Lu, X.-G. Wang, W. Bao, J.-X. Liu, F. Xu, and G.-J. Zhang, *Liquid precursor-derived high-entropy carbide nanopowders*, Ceram. Int. **45**, 22437–22441 (2019), doi:10.1016/j.ceramint.2019.07.244.

[7] X. Yan, L. Constantin, Y. Lu, J.-F. Silvain, M. Nastasi, and B. Cui, $(Hf_{0.2}Zr_{0.2}Ta_{0.2}Nb_{0.2}Ti_{0.2})C$ *high-entropy ceramics with low thermal conductivity*, J. Am. Ceram. Soc. **101**, 4486–4491 (2018), doi:10.1111/jace.15779.

[8] T. Wen, B. Ye, M. C. Nguyen, M. Ma, and Y. Chu, *Thermophysical and mechanical properties of novel high-entropy metal nitride-carbides*, J. Am. Ceram. Soc. **103**, 6475–6489 (2020), doi:10.1111/jace.17333.

[9] L. Feng, W. G. Fahrenholtz, G. E. Hilmas, and Y. Zhou, *Synthesis of single-phase high-entropy carbide powders*, Scr. Mater. **162**, 90–93 (2019), doi:10.1016/j.scriptamat.2018.10.049.

[10] J. Sure, S. S. M. Vishnu, H.-K. Kim, and C. Schwandt, *Facile Electrochemical Synthesis of Nanoscale (TiNbTaZrHf)C High-entropy Carbide Powder*, Angew. Chem. Int. Ed. **59**, 11830–11835 (2020), doi:10.1002/anie.202003530.

[11] J. Gild, K. Kaufmann, K. Vecchio, and J. Luo, *Reactive flash spark plasma sintering of high-entropy ultrahigh temperature ceramics*, Scr. Mater. **170**, 106–110 (2019), doi:10.1016/j.scriptamat.2019.05.039.

[12] J. Zhou, J. Zhang, F. Zhang, B. Niu, L. Lei, and W. Wang, *High-entropy Carbide: A Novel Class of Multicomponent Ceramics*, Ceram. Int. **44**, 22014–22018 (2018), doi:10.1016/j.ceramint.2018.08.100.

[13] P. Sarker, T. Harrington, C. Toher, C. Oses, M. Samiee, J.-P. Maria, D. W. Brenner, K. S. Vecchio, and S. Curtarolo, *High-entropy high-hardness metal carbides discovered by entropy descriptors*, Nat. Commun. **9**, 4980 (2018), doi:10.1038/s41467-018-07160-7.

[14] E. Chicardi, C. García-Garrido, J. Hernández-Saz, and F. Gotor, *Synthesis of all equiatomic five-transition metals High Entropy Carbides of the IVB (Ti, Zr, Hf) and VB (V, Nb, Ta) groups by a low temperature route*, Ceram. Int. **46**, 21421–21430 (2020), doi:10.1016/j.ceramint.2020.05.240.

[15] M. D. Hossain, T. Borman, C. Oses, M. Esters, C. Toher, L. Feng, A. Kumar, W. G. Fahrenholtz, S. Curtarolo, D. Brenner, J. M. LeBeau, and J.-P. Maria, *Entropy Landscaping of High-Entropy Carbides*, Adv. Mater. **33**, 2102904 (2021), doi:10.1002/adma.202102904.

[16] T. J. Harrington, J. Gild, P. Sarker, C. Toher, C. M. Rost, O. F. Dippo, C. McElfresh, K. Kaufmann, E. Marin, L. Borowski, P. E. Hopkins, J. Luo, S. Curtarolo, D. W. Brenner, and K. S. Vecchio, *Phase stability and mechanical properties of novel high entropy transition metal carbides*, Acta Mater. **166**, 271–280 (2019), doi:10.1016/j.actamat.2018.12.054.

[17] K. Kaufmann, D. Maryanovsky, W. M. Mellor, C. Zhu, A. S. Rosengarten, T. J. Harrington, C. Oses, C. Toher, S. Curtarolo, and K. S. Vecchio, *Discovery of high-entropy ceramics via machine learning*, npj Comput. Mater. **6**, 42 (2020), doi:10.1038/s41524-020-0317-6.

[18] E. Chicardi, C. García-Garrido, and F. J. Gotor, *Low temperature synthesis of an equiatomic* $(TiZrHfVNb)C_5$ *high entropy carbide by a mechanically-induced carbon diffusion route*, Ceram. Int. **45**, 21858–21863 (2019), doi:10.1016/j.ceramint.2019.07.195.

[19] X.-F. Wei, J.-X. Liu, F. Li, Y. Qin, Y.-C. Liang, and G.-J. Zhang, *High entropy carbide ceramics from different starting materials*, J. Eur. Ceram. Soc. **39**, 2989–2994 (2019), doi:10.1016/j.jeurceramsoc.2019.04.006.

[20] P. Zhang, X. Liu, A. Cai, Q. Du, X. Yuan, H. Wang, Y. Wu, S. Jiang, and Z. Lu, *High-entropy carbide-nitrides with enhanced toughness and sinterability*, Sci. China Mater. **64**, 2037–2044 (2021), doi:10.1007/s40843-020-1610-9.

[21] O. F. Dippo, N. Mesgarzadeh, T. J. Harrington, G. D. Schrader, and K. S. Vecchio, *Bulk high-entropy nitrides and carbonitrides*, Sci. Rep. **10**, 21288 (2020), doi:10.1038/s41598-020-78175-8.

[22] S. Ma, J. Ma, Z. Yang, Y. Gong, K. Li, G. Yu, and Z. Xue, *Synthesis of novel single-phase high-entropy metal carbonitride ceramic powders*, Int. J. Refract. Metals Hard Mater. **94**, 105390 (2021), doi:10.1016/j.ijrmhm.2020.105390.

[23] D. Liu, H. Liu, S. Ning, B. Ye, and Y. Chu, *Synthesis of high-purity high-entropy metal diboride powders by boro/carbothermal reduction*, J. Am. Ceram. Soc. **102**, 7071–7076 (2019), doi:10.1111/jace.16746.

[24] D. Liu, T. Wen, B. Ye, and Y. Chu, *Synthesis of superfine high-entropy metal diboride powders*, Scr. Mater. **167**, 110–114 (2019), doi:10.1016/j.scriptamat.2019.03.038.

[25] J. Gild, A. Wright, K. Quiambao-Tomko, M. Qin, J. A. Tomko, M. Shafkat bin Hoque, J. L. Braun, B. Bloomfield, D. Martinez, T. Harrington, K. Vec-

chio, P. E. Hopkins, and J. Luo, *Thermal conductivity and hardness of three single-phase high-entropy metal diborides fabricated by borocarbothermal reduction and spark plasma sintering*, Ceram. Int. **46**, 6906–6913 (2020), doi:10.1016/j.ceramint.2019.11.186.

[26] Y. Zhang, Z.-B. Jiang, S.-K. Sun, W.-M. Guo, Q.-S. Chen, J.-X. Qiu, K. Plucknett, and H.-T. Lin, *Microstructure and mechanical properties of high-entropy borides derived from boro/carbothermal reduction*, J. Eur. Ceram. Soc. **39**, 3920–3924 (2019), doi: 10.1016/j.jeurceramsoc.2019.05.017.

[27] L. Feng, W. G. Fahrenholtz, and G. E. Hilmas, *Processing of dense high-entropy boride ceramics*, J. Eur. Ceram. Soc. **40**, 3815–3823 (2020), doi: 10.1016/j.jeurceramsoc.2020.03.065.

[28] L. Feng, F. Monteverde, W. G. Fahrenholtz, and G. E. Hilmas, *Superhard high-entropy AlB_2-type diboride ceramics*, Scr. Mater. **199**, 113855 (2021), doi: 10.1016/j.scriptamat.2021.113855.

[29] J. Gild, Y. Zhang, T. Harrington, S. Jiang, T. Hu, M. C. Quinn, W. M. Mellor, N. Zhou, K. Vecchio, and J. Luo, *High-Entropy Metal Diborides: A New Class of High-Entropy Materials and a New Type of Ultrahigh Temperature Ceramics*, Sci. Rep. **6**, 37946 (2016), doi: 10.1038/srep37946.

[30] G. Tallarita, R. Licheri, S. Garroni, R. Orrù, and G. Cao, *Novel processing route for the fabrication of bulk high-entropy metal diborides*, Scr. Mater. **158**, 100–104 (2019), doi:10.1016/j.scriptamat.2018.08.039.

[31] Y. Zhang, W.-M. Guo, Z.-B. Jiang, Q.-Q. Zhu, S.-K. Sun, Y. You, K. Plucknett, and H.-T. Lin, *Dense high-entropy boride ceramics with ultra-high hardness*, Scr. Mater. **164**, 135–139 (2019), doi: 10.1016/j.scriptamat.2019.01.021.

[32] G. Tallarita, R. Licheri, S. Garroni, S. Barbarossa, R. Orrù, and G. Cao, *High-entropy transition metal diborides by reactive and non-reactive spark plasma sintering: A comparative investigation*, J. Eur. Ceram. Soc. **40**, 942–952 (2020), doi:10.1016/j.jeurceramsoc.2019.10.031.

[33] S. Iwan, K. C. Burrage, B. C. Storr, S. A. Catledge, Y. K. Vohra, R. Hrubiak, and N. Velisavljevic, *High-pressure high-temperature synthesis and thermal equation of state of high-entropy transition metal boride*, AIP Adv. **11**, 035107 (2021), doi:10.1063/5.0045592.

[34] M. Qin, J. Gild, C. Hu, H. Wang, M. S. B. Hoque, J. L. Braun, T. J. Harrington, P. E. Hopkins, K. S. Vecchio, and J. Luo, *Dual-phase high-entropy ultra-high temperature ceramics*, J. Eur. Ceram. Soc. **40**, 5037–5050 (2020), doi:10.1016/j.jeurceramsoc.2020.05.040.

[35] B. Storr, L. Moore, K. Chakrabarty, Z. Mohammed, V. Rangari, C.-C. Chen, and S. A. Catledge, *Properties of high entropy borides synthesized via microwave-induced plasma*, APL Mater. **10**, 061109 (2022), doi: 10.1063/5.0098276.

[36] M. Qin, J. Gild, H. Wang, T. Harrington, K. S. Vecchio, and J. Luo, *Dissolving and stabilizing soft WB_2 and MoB_2 phases into high-entropy borides via boron-metals reactive sintering to attain higher hardness*, J. Eur. Ceram. Soc. **40**, 4348–4353 (2020), doi: 10.1016/j.jeurceramsoc.2020.03.063.
